# Snake venom toxins in cancer therapy: advances in targeted delivery and peptide design

**DOI:** 10.1007/s44446-026-00116-9

**Published:** 2026-10-26

**Authors:** Akmal M. Asrorov, Matluba Mirzaeva, Muzaffar Kayumov, Shamsiddin Fazliddinov, Khamza S. Toshov, Okhunjon Khasanov, Gulnora Rakhmonova, Ahmidin Wali, Hayotbek A. Abdullaev, Nurkhodja Mukhamedov

**Affiliations:** 1 Institute of Bioorganic Chemistry, Uzbekistan Academy of Sciences, 83, M. Ulughbek Street, 100125 Tashkent, Uzbekistan; 2 Pharmaceutical Technical University, Tashkent BioPharmaCity, 1 Bogzor Street, Tashkent, Uzbekistan; 3 Zhongshan Institute for Drug Discovery, Shanghai Institute of Materia Medica, CAS, 528400 Zhongshan, China; 4 Tashkent State Medical University, 2, Farabiy Street, 100109 Tashkent, Uzbekistan; 5 National University of Uzbekistan, 4, Universitet Street, 100174 Tashkent, Uzbekistan; 6 Kimyo International University, 156, Rustaveli Street, 100121 Tashkent, Uzbekistan; 7 Xinjiang Technical Institute of Physics and Chemistry, 40-1 Beijing Road, 830011 Urumqi, China; 8 National Biopharmaceutical Research Institute, Tashkent BioPharmaCity, 1 Bogzor Street, Tashkent, Uzbekistan

**Keywords:** Snake venom, Cancer therapy, Disintegrin, Cilengitide, Delivery systems, Peptides

## Abstract

Snake venom represents a potent biological arsenal of cytotoxic compounds. Venom proteins and peptides, including disintegrins, venomous enzymes, three-finger toxins, and serine proteases, have garnered attention as effective anticancer agents. Despite their efficacy at nano- or micromolar concentrations, non-standardized mixtures often limit their therapeutic utilization, necessitating fractionation to minimize side effects. This review examines the antitumor potential of snake venom and its constituent proteins and peptides through a literature search in NCBI PubMed. We highlight the antitumor efficacies of venom polypeptides by correlating them with IC_50_ values across various cell lines, demonstrating micromolar cytotoxic concentrations. The review encompasses several key areas: whole venom antitumor efficacy, mechanisms of action of individual proteins and peptides, fractionation and purification methods, cytotoxic effects of low molecular weight peptides, and delivery system approaches utilizing peptide sequences as cell-penetrating mechanisms alongside liposomes and nanoparticles for enhanced polypeptide toxicity. Given that cilengitide contains an RGD sequence analogous to disintegrins and targets integrins, we provide a dedicated analysis of its anticancer potential and clinical trial outcomes. Clinical evidence indicates the necessity for further investigation into the synergistic effects of cilengitide with established chemotherapeutic agents. The scientific data reviewed demonstrate that antimetastatic properties constitute the most extensively studied mechanism of venom polypeptides. This review underscores the therapeutic promise of venom-derived polypeptides while emphasizing the critical need for advanced delivery systems and rigorous clinical validation to translate these findings into viable cancer therapeutics.

**Graphical Abstract:**

**Figure:**
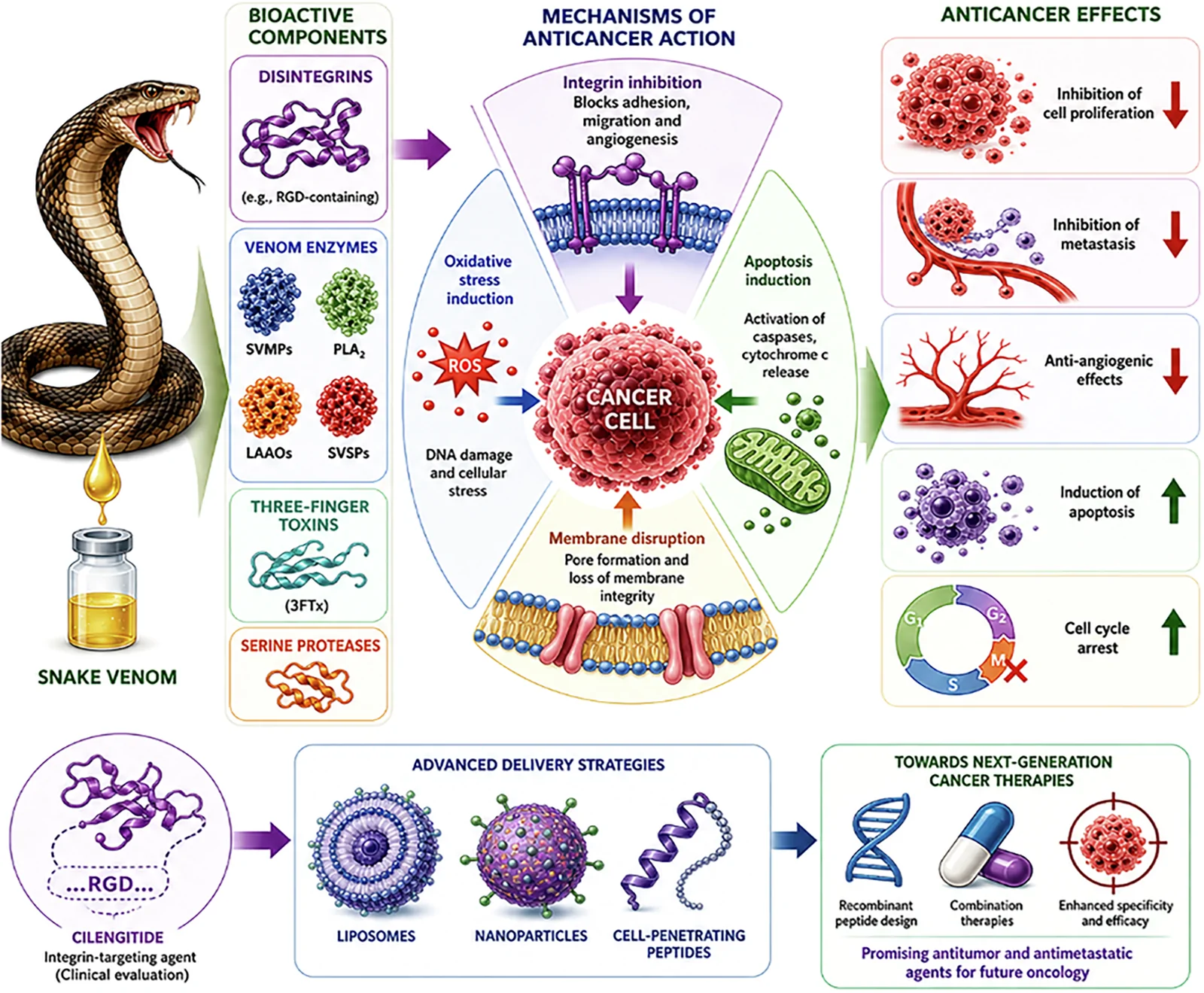

## Highlights

- Snake venom peptides exhibit strong and selective anticancer activity.
- Disintegrins target integrins to inhibit metastasis and angiogenesis.
- Venom enzymes induce apoptosis via oxidative stress mechanisms.
- Cytotoxins disrupt membranes and activate caspase-dependent pathways.
- Recombinant and combinatorial strategies enhance therapeutic potential.

## Introduction

The central challenge in oncology is not only killing cancer cells effectively but also preventing metastasis and overcoming drug resistance. Cancer remains one of the leading causes of mortality worldwide, with conventional therapies often proving ineffective in advanced stages and metastatic disease. It is a growing global health challenge that deserves special attention. In 2022 alone, approximately 20 million new cases were diagnosed, with 9.7 million deaths worldwide (Bray et al. 2024). Approximately one in nine men and one in twelve women die of cancer, respectively (Bray et al. 2024). These figures can be significantly greater due to unregistered cases in developing regions. Although dozens of effective anticancer drugs are available in medical practice (Andersson et al. 2023), they may not be effective for patients in the late stages of cancer. Therefore, surgery is a prerequisite to extending patients’ lives. However, the surgery carries a risk of metastasis (Tohme et al. 2017), which must be mitigated. Thus, biological agents having antitumor potential and cell adhesion-inhibiting properties are of considerable interest. In this aspect, snake venom is one of the alternatives that deserves attention. Natural products represent a rich source of diverse bioactive compounds for drug discovery. Among them, snake venom emerges as a reservoir of cytotoxic, anti-angiogenic, and anti-metastatic proteins and peptides. Unlike conventional chemotherapeutics that target cancer cells to a limited extent, venom-derived toxins have evolved to recognize specific biomolecules (integrins, ion channels, membrane phospholipids) with high affinity.

Snake venom possesses a wide range of pharmacological activities applicable to various diseases. It is a cocktail of various higher and lower molecular substances that show cytotoxic effects. Proteins and peptides have significant contribution to the cytotoxicity of the venom. Several therapeutics have been derived from snake venom, including serine proteases, disintegrins, metalloproteinases, hypotensive peptides, antivirals, mitogenic substances, and those possessing antinociceptive or fibrinolytic activities (Waheed et al. 2017). Various enzymes in snake venoms, such as proteases and kinases, have been investigated for their cytotoxic effects (Vyas et al. 2013, Uzair et al. 2018). Therefore, these enzymes warrant further investigation as potential anticancer agents. Proteins and peptides are the major class of compounds that reveal cytotoxic effects (Diniz-Sousa et al. 2023, Bedraoui et al. 2024). Snake venom is a rich source of peptides with antitumor and antimicrobial properties (Pérez-Peinado et al. 2020). Various peptides in snake venoms reveal antiproliferative activities in cancer cells. The authors also suggested the high pharmacological potential of snake venom due to their peptides (de Moura et al. 2022).

**Fig. 1:**
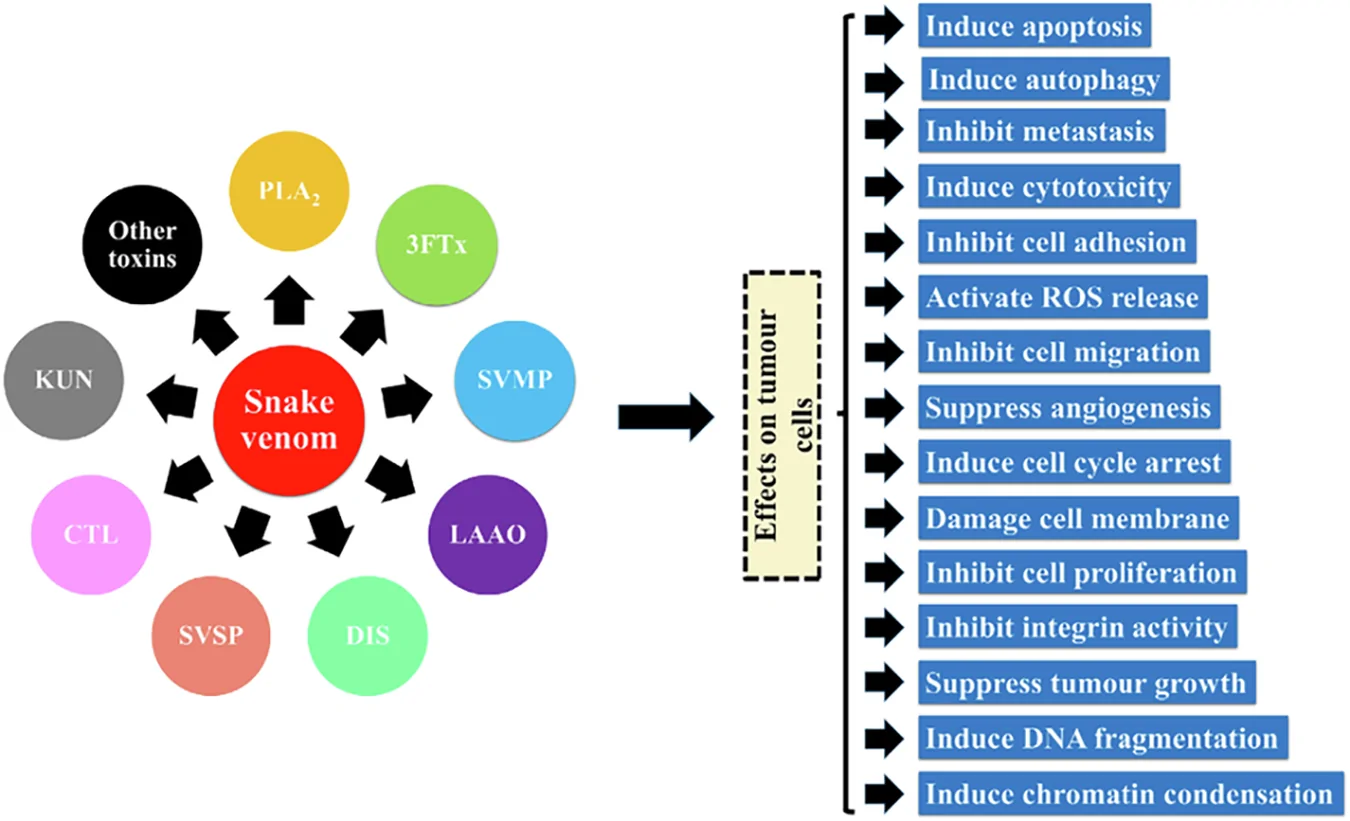
Toxic polypeptides of snake venom and their effects on cancer cells. The abbreviations of three-finger toxin (3FTx), C-type lectin (CTL), disintegrin (DIS), Kunitz-type serine protease inhibitor (KUN), L-amino acid oxidase (LAAO), phospholipase A2 (PLA2), snake venom metalloproteinase (SVMP), and snake venom serine protease (SVSP) are given on the left side of the figure. The figure has been reused from (Offor and Piater 2024) with the permission of Wiley and Sons

Venom proteins, independent of whether they cause enzymatic or non-enzymatic actions on cancer cells, mainly lead to apoptosis. Various mechanisms occur with the venom proteins, leading to programmed cell death (Ejaz et al. 2018). The molecular mechanisms of snake venom toxins include altered levels of apoptotic machinery leading to genome instability, interactions with integrins, reduction of epithelial-mesenchymal transition, inhibition of cell cycle progression, inhibition of DNA damage repair enzymes, reduction in the level of proteinases, etc. (Fig. 1) (Estevão-Costa et al. 2018, Offor and Piater 2024). These and other outcomes result from various molecules. Elucidating the molecular mechanisms of individual peptides or at least fractions varying based on their molecular weights can detect new avenues to understanding and utilizing snake venom in medicine. In this aspect, snake venom can be an appropriate source since it contains various molecules revealing cytotoxic effects and numerous molecules binding integrins.

Although snake venom contains numerous proteins and peptides with high cytotoxicity, data on their antitumor activity are limited because they are present in low amounts. On the other hand, non-standardized mixtures in venomous compounds often cause highly deviating results because of reasons such as the compositions of venoms taken in different seasons of the year vary a lot. We can observe varying compositions of venoms depending on the geographical locations of snakes. In a separate section, named ‘Fractionation and purification’, we discussed obtaining fractions and purification of polypeptides that have been deserving special attention in this discipline of research.

This review aims to comprehensively examine the antitumor potential of snake venom-derived polypeptides, including disintegrins, three-finger toxins, phospholipases, L-amino acid oxidases, and serine proteases, by discussing their in vitro and in vivo outcomes as well as challenges to translation to clinical practice. In this review, we reviewed the anticancer potential of proteins and peptides of snake venom origin, from the discovery of disintegrins as integrin blockers to the clinical results of cilengitide. We also explored advances in venom fractionation, peptide delivery systems, and the clinical trajectory of an integrin-targeting analog termed cilengitide, highlighting both promises and hurdles in harnessing venom toxins for oncotherapy.

Several comprehensive reviews have summarized the anticancer potential of snake venom toxins. Differing from the previously published reviews (Li et al. 2018, Akhtar et al. 2021, Oliveira et al. 2022, Offor and Piater 2024), in our current work, we paid special attention to three significant points. First, we organized discussions around the *‘barrier-to-solutions’* framework by explicitly linking the biological limitations of venom toxins, such as micromolar efficacy, systemic toxicity, and venom heterogeneity, to the engineering strategies designed to overcome them, including fractionation, peptide designing, encapsulation in nanoparticles or liposomes, and conjugating with cell-penetrating peptides. Second, we provided a comprehensive, critical analysis of cilengitide as a translational case study that extracts actionable conclusions from clinical trials for future venom-inspired drug development. Third, we discussed secondary structures of polypeptides from venom origin, an area overshadowed by protein-focused reviews. Collectively, these features help readers to better analyze the outcomes in this discipline obtained in the last fifteen-sixteen years.

## Methods

We conducted a literature search in the NCBI PubMed database to identify studies on snake venom and its antitumor potential from 2010 to 2025. References were selected based on their relevance to anticancer and antitumor activities. In the virtual search, the other used keywords were ‘*the toxin name* + *antitumor potential*’ in PubMed. We also used a few additional earlier published PubMed references to provide more accurate interpretations of the data used. The search was confined to NCBI PubMed to ensure the most reliable consistency, given the vast volume of literature available in other databases. This methodological approach ensured a comprehensive review of venom-derived anticancer agents and their mechanisms.

The screening of the data was carried out by analysing the titles and abstracts of the references downloaded from PubMed as a single text file based on ‘*best match*’ sorting. The contents of the selected papers were further studied in their full forms. The studies that did not study either antitumor or antiproliferative effects were excluded. We used the Figures from the original sources by obtaining formal permission from the corresponding publisher.

in vitro and in vivo outcomes of disintegrins were provided in a table format to ease the comparison of their antitumor potential-related data (Table 1). The polypeptide name, the source of origin, MW, IC_50_ values, cell types, and the results were provided. We summarized the advantages and disadvantages of methods of chromatography used to fractionate and isolate toxins from snake venoms (Table 2). The clinical outcomes of cilengitide, holding a cyclic RGD peptide derived from disintegrins, have been summarized in Table 3. The number of patients, their median age, the maximum dose, the treatment duration, toxicity, and other relevant data were included.

**Table 1:** Disintegrins and their anticancer potential-related results

Disintegrin	Origin	MW	IC_50_ values	Cell type	Results	Ref.
**Adinbitor**	*Agkistrodon halys brevicaudus stejneger*	9 kDa	0.89 µM	bFGF-induced ECV304 cells	The reduced level of angiogenesis was explained as the main cause for the revealed anticancer efficacy of adinbitor.	(Wang et al. 2004)
			25.5 µM	SMMC-7721 cells	The formation of apoptosis by adinbitor was linked with binding integrins. Effects were similar to 5-FU at a 100 μg/mL dose.	(Zhao et al. 2008)
**Viridistatin 2**	*Crotalus viridis viridis*	7.85 kDa		T24, SK-MEL-28, MDA-MB-231	At a 6.3 µM concentration, it inhibited the invasion of T24, SK-MEL-28, and MDA-MB-231 cells 3–5 times. The migration of these cells for 3 h was only ≤20% in the presence of viridistatin. Its 1 mg/kg administration reduced the growth of B16F10 tumors in mice threefold.	(Lucena et al. 2012)
			10.6 µM	BXPC-3 cells	Its effects on BXPC-3 pancreatic cancer cells were fourfold greater than paclitaxel but not doxorubicin. Its combination with mojastin 1 did not result in a synergistic effect.	(Lucena et al. 2015)
**Mojastin 1**	*Crotalus scutulatus scutulatus*	7.4–7.6 kDa		T24, SK-MEL-28	At a 6.3 µM, it inhibited the invasion of T24, SK-MEL-28, and MDA-MB-231 cells 3–7 times compared to PBS treatment.	(Lucena et al. 2012)
			8.7 µM	BXPC-3 cells	Its effects on BXPC-3 pancreatic cancer cells were >four-fold greater than paclitaxel but not doxorubicin. The combination of mojastin 1 and viridistatin 2 did not result in a synergistic effect.	(Lucena et al. 2015)
**Tzabcanin**	*Crotalus simus tzabcan*	7.1 kDa	0.75 µM >2 µM	A375 cells A549 cells	It was reported to reduce the adhesion of A375 melanoma and A549 adenocarcinoma cells because it interacts with α_v_β_3_. A 1 µg/µl of Tzabcanin significantly reduced the migration levels of A375 and A549 cells. Tzabcanin showed greater effects on A375	(Saviola et al. 2016)
**Leucurogin**	*Bothrops leucurus*	10.4 kDa		Ehrlich tumor	Its 5 µg daily administration significantly reduced Ehrlich tumor growth in mice. A 10 µg administration halved the tumor mass, and a 20 µg administration reduced the tumor volume to one-third of the control saline level.	(Higuchi et al. 2011)
**Contortrostatin**	*Agkistrodon contortrix contortrix*	13.5 kDa		MDA-MB-435	A 30 µg daily administration to a mouse was well tolerated and resulted in a 3–4 times smaller MDA-MB-435 tumor volume for 3–6 weeks. Contortrostatin was suggested to inhibit angiogenesis by blocking αvβ3.	(Zhou et al. 1999)
				MDA-MB-435	Its administration at 5 µg per day inhibited tumor growth in mice bearing MDA-MB-435 breast cancer cells by >65%. It prevented its metastasis and angiogenesis.	(Zhou et al. 2000)
**Vicrostatin**	*Agkistrodon contortrix contortrix*	7.15 kDa		U251	Radioiodinated vicrostatin (^131^I-VCN), consisting of 69 amino acids, showed efficacy against glioblastoma. The iodinated protein extended the total survival rate of mice bearing glioma by >10 days. The 50% survival rate was extended by >20 days.	(Swenson et al. 2018)
				SKOV3	A weekly one-time administration of 5 µg vicrostatin to mice bearing SKOV3 cells showed higher efficacy than its two-time administrations at half doses. The protein doubled the survival rate of mice bearing U87 cells. Similar results were observed in U251-bearing mice.	(Swenson et al. 2020)
**Mictlan D3**	*Crotalus mictlantecuhtli*	7.44–7.51 kDa		MDA-MB-231	A 16 µM of mictlan D3reduced the MDA-MB-231 cell viability level by <70%. Its 1 µM reduced the level of cell adhesion to fibronectin and laminin by >60 and >80%, respectively. Similarly, >60% inhibition of cell migration was observed following the treatment with a 1 µM of mictlan-D3.	(Rivas-Mercado et al. 2025)
**Alternagin-C**	*Bothrops alternatus*	29 kDa		MDA-MB-231	At a 1 µM concentration, it halved the attachment of MDA-MB-231 cells to collagen. It stimulated the expression of metastasis suppressor 1 (MTSS1). The authors suggested its efficacy against triple-negative breast cancer.	(Moritz et al. 2022)
**Lansbermin-I**	*Porthidium lansbergii lansbergii*	<10 kDa		MCF-7 MDA-MB-231	It suppressed the adhesion and migration of MCF-7 and MDA-MB-231 breast cancer cells in vitro. It revealed no effects on nontumor breast cells. The antitumor efficacy of the protein was attributed to the RGD interaction with integrins containing α2 and/or β1 units	(Montealegre-Sánchez et al. 2019)
**Lebein**	*Macrovipera lebetina*	6.99 kDa		LS174, HCT116, HT29	At a 0.2 µM, it suppressed the cell viability levels of LS174, HCT116, and HT29 cancer cells several-fold. Linear changes were observed at higher concentrations up to 1 µM. The highest effects were observed in HCT116 cells, which were linked with caspase-independent apoptosis. Induction of caspase-3 and -8 and PARP cleavage were the main reasons for cytotoxicity in LS174 cells.	(Zakraoui et al. 2017)
				SK-MEL-28	It revealed great cytotoxic efficacy in melanoma cells. At 10 nM, it suppressed the viability levels of SK-MEL-28 and LU-1205 cells three- and five-fold, respectively. The inductions of ROS and p53 were suggested as the main reasons. The apoptosis was found to be caspase-independent	(Hammouda et al. 2016)
**Jarastatin**	*Bothrops jararaca*	7.75–7.76 kDa			It binds αvβ3, causing the anticancer effect. The molecular dynamics studies revealed an open/closed conformational equilibrium in integrins. Based on computational calculations, the authors concluded that disintegrins can either be the antagonist of integrin function or its agonist/partial agonist.	(Vasconcelos et al. 2024)

**Table 2:** Comparison of methods for fractionation and isolation of snake venom toxins

Method	Advantages	Disadvantages
Size-Exclusion Chromatography (SEC)	- Gentle on proteins - Separates by size - Good for native protein complexes	- Low resolution for similarly sized proteins - Time-consuming - Not ideal for peptides
Ion-Exchange Chromatography (IEX)	- Separates based on charge - Scalable - Good for fractionating venom toxins	- Sensitive to buffer conditions - Not ideal for neutral molecules - Requires desalting
Affinity Chromatography	- High specificity - Efficient purification if the ligand is available	- Requires a known ligand - Expensive and sometimes hard to optimize
RP-HPLC	- High resolution - Good for hydrophobic peptides - Widely used in proteomics - Compatible with MS	- Poor separation of polar compounds - Can cause peptide denaturation - Requires organic solvents
Hydrophilic Interaction Liquid Chromatography	- Good for small polar molecules and glycopeptides - MS-compatible - Complements RP-HPLC	- Less effective for hydrophobic proteins - Complex retention behavior - May require desalting
Gel Electrophoresis	- High resolution - Visual confirmation of size - Inexpensive.	- Labor-intensive - Not quantitative - Limited preparative scale
Off-Gel Electrophoresis	- Good separation by isoelectric point - Can collect fractions for MS	- Expensive and time-consuming - Limited to peptides/proteins with clear pI differences
Ultrafiltration	- Simple and fast - Good for preliminary separation by size	- Low resolution - Can lead to loss of proteins - Non-specific

**Table 3:** The outcomes of cilengitide in clinical trials against tumors of various organs

Clinical trial	Cancer type	Years of clinical trials	Number of patients	Median age of patients	Maximal dose of cilengitide	Treatment duration	Toxicity	Result of the study	Ref
Phase I	Various solid tumors	2003–2008	35	56	2000 mg/2× week	4 weeks	Low	No relationship was observed between the change in tumor size and the dose of cilengitide	(O’Donnell et al. 2012)
Phase II	Newly diagnosed glioblastoma	2005–2017	112	55.5	2000 mg/week	4 weeks	High	The combination of cilengitide with chemoradiation can extend the life expectancy of GBM patients	(Nabors et al. 2012)
Phase II	Non-metastatic prostate cancer	2005–2007	16	65.5	2000 mg/2× week	4 weeks	High	No detectable clinical outcomes and questionable antimetastatic effect as a single agent	(Alva et al. 2012)
Phase II	Metastatic melanoma	2004–2006	26	61	2000 mg/2× week	4 weeks	High	Minimal clinical efficacies were observed in patients by a 2000 mg dose given twice a week	(Kim et al. 2012)
Phase II	Recurrent glioblastoma	No data	30	54	2000 mg/2× week	Maximum 2 years	High	A two-time cilengitide given at 2000 mg doses was tolerated, and no dose reduction was required	(Gilbert et al. 2012)
Phase I	Stage III of non-small lung cancer	2010–2013	14	62.5	80 mg/m^2^	3 months	High	The combination of cilengitide with radiotherapy resulted in encouraging clinical results	(Massabeau et al. 2018)
Phase II	Advanced non-small lung cancer	1999–2001	140	59.3	600 mg/m^2^	2 months	Low	Similar one-year survival rates were observed by 600 mg/m^2^ cilengitide and 75 mg/m^2^ of docetaxel	(Manegold et al. 2013)
Phase II	High-grade glioma	2008–2010	30	14.2	1800 mg/m^2^	Maximum 20 months	Low	The combination of cilengitide with radiation and chemotherapy resulted in a synergistic effect.	(MacDonald et al. 2013)
Phase II	Metastatic head and neck squamous cell carcinoma	2008–2010	182	58	2000 mg/2× week	Unknown	High	A one- or two-time cilengitide administration did not improve the effects of cetuximab	(Vermorken et al. 2014)
Phase I	Various solid tumors	2011–2012	12	56	2000 mg/2× week	Unknown	Low	A one- or two-time cilengitide administration did not enhance the effects of 90 mg/m^2^ of paclitaxel	(Haddad et al. 2017)
Phase I	Recurrent glioblastoma	2010–2011	45	≥18 years Old	2000 mg/2× week	22 months	Low	The administration of weekly two-time cilengitide and daily mg of cediranib did not improve the survival rate	(Gerstner et al. 2015)
Phase II	Non-small cell lung cancer	No data	220	58.6	2000 mg/2× week	24 months	Low	The combination of cilengitide with cetuximab/ chemotherapy indicated clinical activity	(Vansteenkiste et al. 2015)

In the review paper, we used 14 Figures: ten of them from Fig. 1 to 10 were reused from the original already published sources with the permissions of the publishers due to their originality; 4 of them from Fig. 11 to 14, belonging to the secondary and tertiary structures of snake venom polypeptides were generated by ourselves, and published for the first time.

## Antitumor effectiveness of snake venoms

Crude snake venoms have long been recognized as complex biological cocktails containing hundreds of bioactive molecules, yet their evaluation as anticancer agents reveals contradictory results. On one hand, venoms exhibit undeniable cytotoxicity against cancer cells; on the other hand, their inherent heterogeneity complicates mechanistic interpretation and clinical translation. Unlike purified pharmaceutical agents, crude venoms vary not only across species but also within the same species due to seasonal, geographical, and even individual physiological factors. Nevertheless, the consistent observation of selective cytotoxicity across diverse cancer cells provides compelling justification for continued investigation. In this section, we evaluated anticancer activities of crude snake venoms, paying particular attention to methodological disparities, IC_50_ values on various cancer cells, selectivity on various cells, and molecular mechanisms, where consistent.

Crude snake venoms exhibit selective cytotoxicity against cancer cell lines compared to normal cells. In one work, *Vipera anatolica* venom was found to show greater toxic effects on U87MG human glioblastoma cells over HEK293 non-cancerous cells with IC_50_ values of 0.75 and 5.52 µg/mL, respectively. Several other research works have demonstrated the antitumor properties of snake venom. Kerkkamp et al. (2018) reported anticancer properties of snake venom in PaTu 8988t pancreatic cancer cells. The IC_50_ values of venoms isolated from *Ophiophagus hannah*, *Echis carinatus*, *Naja annulifera*, and *Naja kaouthia* were similar and ranged from 1.39 to 2.09 ng/mL. Their IC_50_ values in ZF4 zebrafish fibroblast cells were at least twenty-fold greater (Kerkkamp et al. 2018). While the reported IC_50_ values in the ng/mL range are remarkably potent and surpass those of many conventional chemotherapeutics, several caveats warrant careful consideration. The twenty-fold smaller cytotoxicity for cancer cells over ZF4 zebrafish fibroblasts is encouraging; however, zebrafish fibroblasts represent a distant model from human normal tissues. Testing against human fibroblasts and epithelial cells can provide a more consistent comparison.

Most of the venoms or their compounds reveal cytotoxic or anticancer potentials in their µg/mL or micromolar concentrations. For example, the effects of *Vipera lebetina turanica* venom toxin on two cervical cancer cells showed its potential at <10 µg/mL. Its IC_50_ values in Ca Ski and C33a cervical cancer cells were 8.77 and 6.59 µg/mL, respectively. A 12 µg/mL concentration enhanced the apoptosis rate by > 87% in these cells. Administration of 1 mg/kg to mice reduced tumor xenografts by 65% compared to the control. The results show similar anticancer efficacies of *Vipera lebetina turanica* venom toxin on two Ca Ski and C33a cervical cancer cells that were linked with the overexpression DR3 and DR5 death receptors, and inactivation of NF-kB (Lee et al. 2016). The authors claimed the anticancer efficacy of the venom toxin against cervical cancer or chemoresistant breast cancer cells.

In another work, the anticancer efficacy of *Vipera lebetina turanica* venom on ovarian cancer cells was studied. Its IC_50_ values in PA-1 and SK-OV3 cells were 4.5 and 6.5 μg/mL, respectively. The occurring apoptosis was linked with the induction of Bax and caspase-3 proteins. Besides, the suppression of NF-κB and DNA binding of STAT3 was detected following the treatment. The authors suggested identifying active molecules for further therapeutic development (Song et al. 2012). The snake venom *Vipera raddei kurdistanica* was reported to be effective against breast cancer types. The venom showed its IC_50_ values in MCF-7, MDA-MB-231, and MCF-10a breast cancer cells were 18.5, 20.3, and 243.4 µg/mL for 24 h, which lowered to 0.98, 1.29, and 37 µg/mL for 96 h. The venom resulted in significant changes in ROS and glutathione amounts (Malekara et al. 2020). These results show several-fold differences in the effects of *Vipera raddei kurdistanica* on various breast cancer cells. Mechanistic studies can clarify the reasons for several-fold or several-ten fold differences.

**Fig. 2:**
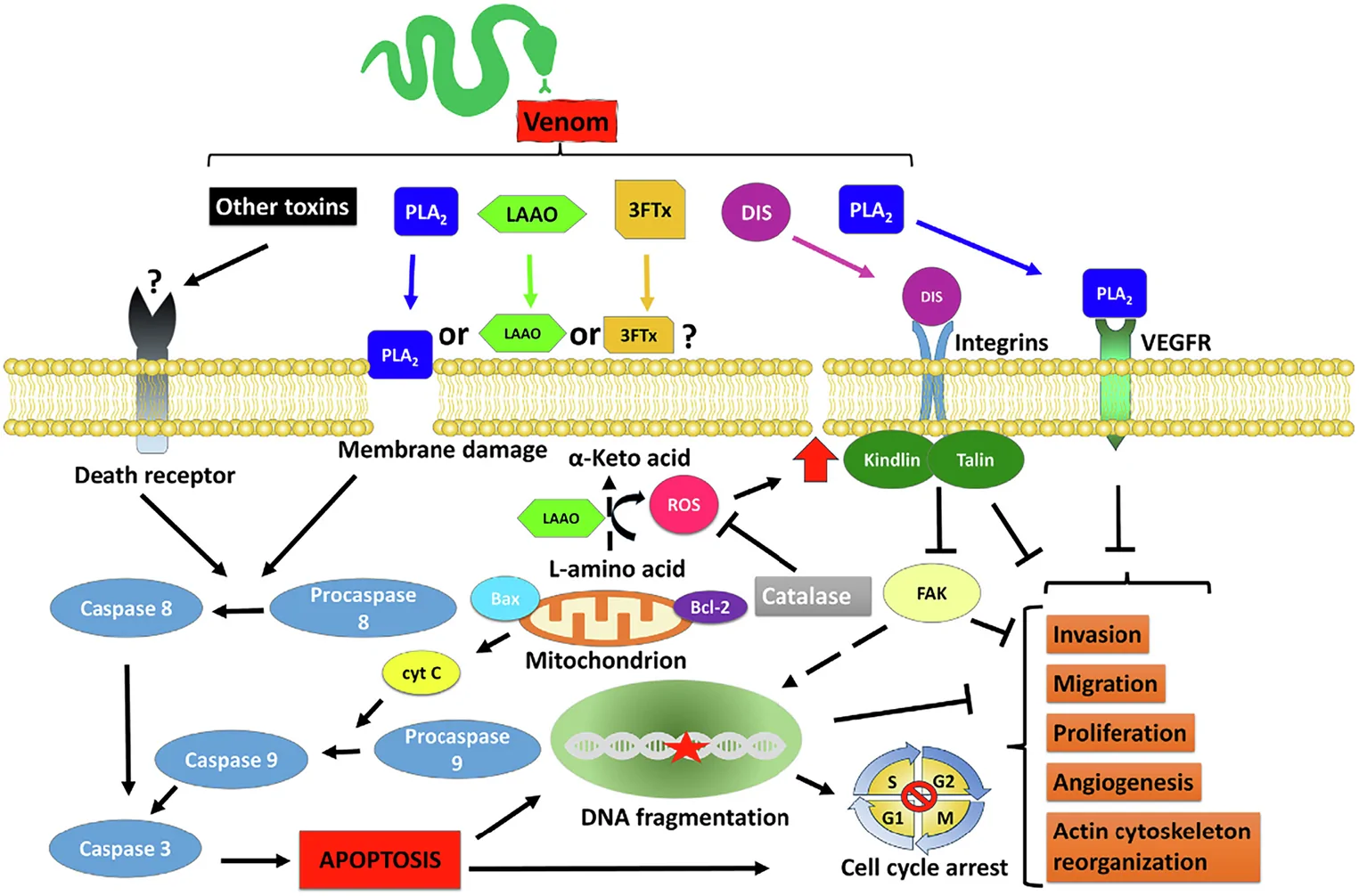
Mechanisms of action of snake venom causing cytotoxicity. The figure has been reused from (Offor and Piater 2024) with the permission of Wiley and Sons

Certain venom proteins, such as PLA2, LAAO, and/or 3FTx, induce membrane disruption and apoptosis, whereas disintegrins target integrins, leading to cytoskeletal reorganization. Some other members, such as LAAO, lead to the alteration of ROS and cause an imbalance in the oxidoreductive system. PLA2 inhibits metastasis and cell migration by inhibiting VEGF (Fig. 2) (Ejaz et al. 2018). The drugs developed based on snake venoms that reached medical practice or clinical/preclinical trials target angiotensin-converting enzyme, glycoprotein IIb/IIIa, fibrinogen, prothrombin, thrombin, natriuretic peptide receptor, nicotinic acetylcholine receptor, and integrin receptor. The anticancer potential of snake venom is linked with three general processes: membrane disruption, interference with the synthesis of proteins and nucleic acids, and activation of immune-cell interactions (Pérez-Peinado et al. 2020).

The whole snake venom can be concluded to possess significant cytotoxicity against cancer cells, which is expected to be more than against normal cells. These results indicate that it is a rich source of natural biologicals, worthy of fractionation and purification for further development. These findings justify the continued exploration of snake venom as a rich source of antitumor compounds. The limitations inherent to whole venom preparations, particularly their heterogeneity, batch variability, and the confounding effects of synergistic toxicity, strongly support the rationale for isolating and characterizing individual venom components. While whole venoms provide a valuable source for initial screening, the true therapeutic potential lies in their constituent proteins and peptides, which can be purified, structurally characterized, and mechanistically investigated. In the following sections, we examine the anticancer properties of the isolated components, starting with integrin-binding proteins and peptides, the class of venom toxins that can progress toward clinical application.

## Integrin-binding proteins and peptides

Integrin-binding proteins and peptides, particularly the disintegrin family, represent the most intensively investigated and therapeutically promising class of snake venom-derived anticancer agents. Integrins are transmembrane receptors that mediate cell-extracellular matrix adhesion, migration, proliferation, and survival, making them attractive targets for antimetastatic and anti-angiogenic intervention. Disintegrins, possessing high affinity to the extracellular domains of integrins, function as cancer-inhibiting molecules. In this section, we review the anticancer activities of disintegrins, paying special attention to their structure- and MW-related features as well as their in vitro results.

**Fig. 3:**
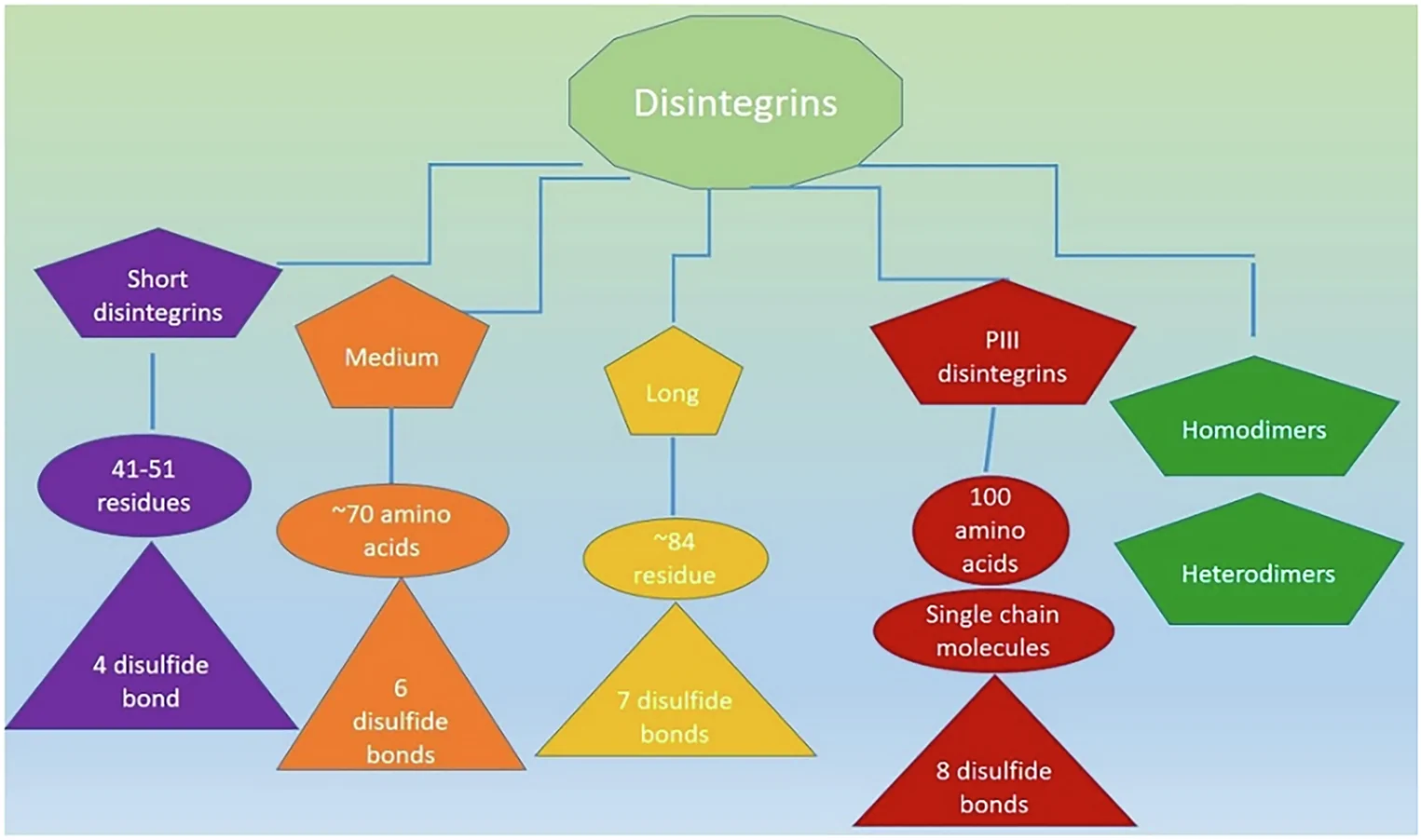
Types of disintegrins and varying disulfide bonds held. The figure has been reused from (Akhtar et al. 2021) with the permission of Elsevier

Snake venom-derived disintegrins and integrin-binding peptides critically inhibit cancer cell adhesion, migration, and angiogenesis. Antitumor activities of several proteins isolated from snake venom have been established. In most cases, their anticancer potentials were linked with the prevention of angiogenesis and the induction of apoptosis (Li et al. 2018). Disintegrins play a central role in this mechanism. They are proteins that inhibit integrins, playing essential roles in cell migration and cell adhesion. They vary significantly in their molecular weights. Anticancer efficacies of disintegrins were proven in various cancer cells. There are four, six, and seven disulfide bonds in short, medium, and long disintegrins, respectively. Eight disulfide bonds and 100 amino acid residues are present in disintegrins, termed PIII (Fig. 3) (Akhtar et al. 2021). In this section, we discuss the anticancer efficacies of various polypeptides that bind integrins.

The diversity and classification of disintegrins show their range from short variants with 4–5 kDa MW to long ones with up to 12–13 kDa (Fig. 3). The effects of disintegrins have been established on several cancer types (Schönthal et al. 2020). The inhibition of integrins such as αvβ3, αvβ5, and αvβ8 expressed in glioblastoma blood vessels and tumor cells can contribute to tumor suppression in glioblastoma patients (Roth et al. 2013). Integrins are involved in cell adhesion of breast cancer (Mirzaeva et al. 2026), which is the most common cancer among women (Mirzaeva et al. 2025). However, significant challenges arise in finding a linkage between their therapeutic efficacy and MWs. On the other hand, the multiplicity of isoforms, often co-expressed in the same venom, complicates the isolation of individual species and raises questions about functional redundancy. High structural similarity of disintegrins necessitates rigorous functional characterization in terms of finding a correlation between amino acid sequences and functions.

**Fig. 4:**
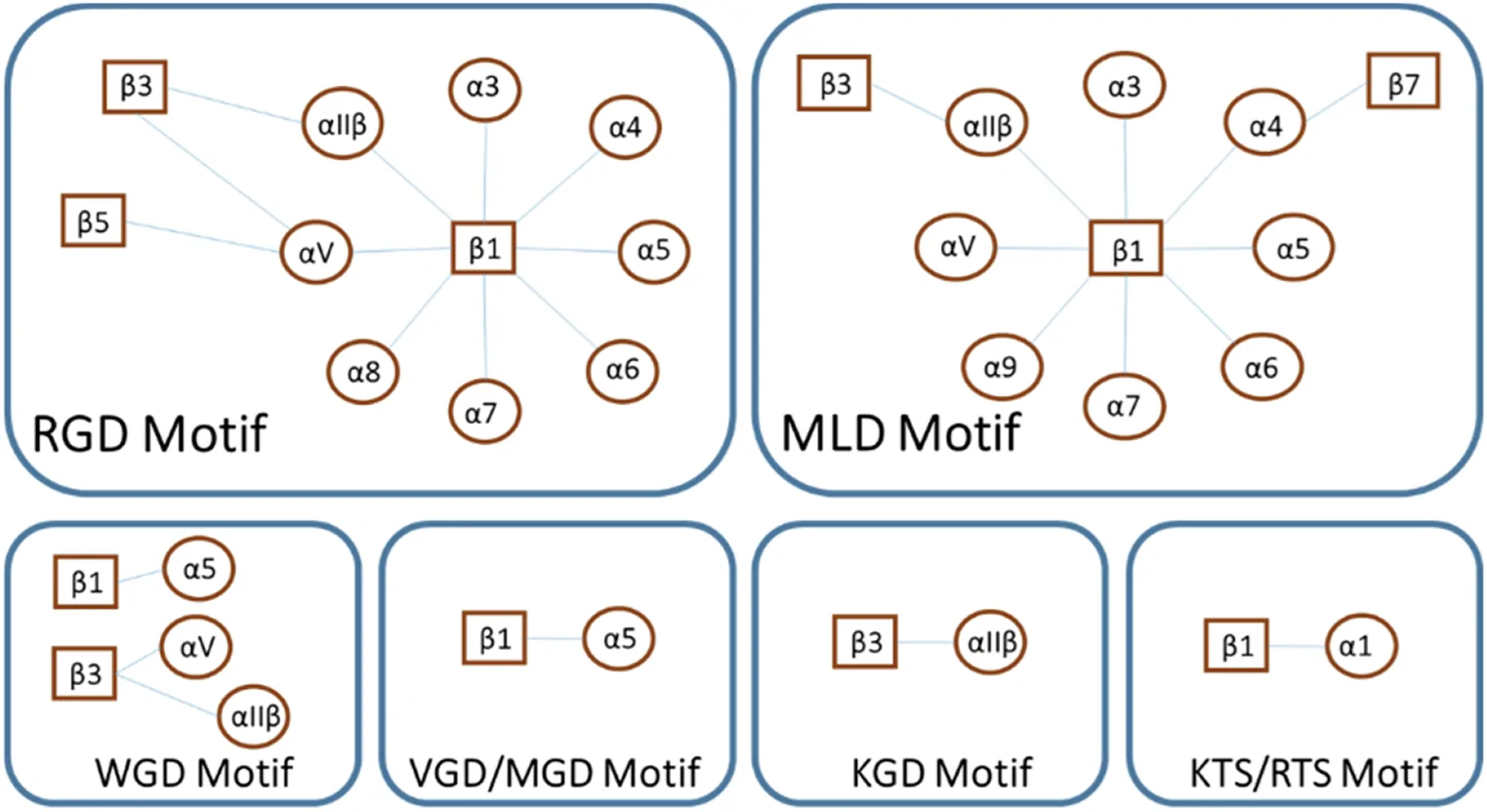
Amino acid motifs occurring in disintegrins and their interactions with various types of integrins. Circles were used for α subunits, and squares stand for β subunits. The Figure has been reused from (Kolvekar et al. 2023) with the permission of Elsevier

The ligands involved in interactions with integrins, derived from snake venoms, are laminin, fibronectin, thrombospondin-1, VEGF-A, vascular cell adhesion protein 1, angiostatin, osteopontin, tissue-type transglutaminase, tenascin-C, vitronectin, intercellular adhesion molecule 1, nephronectin, and others. Many motifs are involved in these interactions between disintegrins and integrins that prevent cell adhesion and migration (Fig. 4) (Kolvekar et al. 2023). In many of these interactions, the role of the RGD motif is suggested to be a significant part of binding integrins like αIIbβ3 and αvβ3. The interaction with RGD was concluded to lower the number of attractive bonds with *BRAF*V600E (Bialves et al. 2024), playing a significant role in the cell migration of cancerous cells (Śmiech et al. 2022). The KGD sequence (Arg substituted with lysine) is expected to have specificity to αIIbβ3 (Arruda Macêdo et al. 2015).

Integrin α9β1 was reported to be a receptor for nerve growth factor (NGF) (Staniszewska et al. 2008). A high anticancer efficacy of this polypeptide was shown by (Osipov et al. 2014). An 8 nM/kg administration of NGF isolated from cobra venom was found to reduce the growth of Ehrlich ascites carcinoma for 13 days, from day 19 to day 32 after treatment. The effect resulted from the repeated drug administration every 3–4 days. The authors linked the tumor volume reduction with the TrkA receptor. The effect of the protein was found to be short-term and loses its effect as the drug administration stops (Osipov et al. 2014).

Besides RGD, KTS was reported as another disintegrin motif targeting α1β1 integrin. The integrins αvβ3, αvβ5, αvβ6, and α5β1 were targets in HBMEC cells and chicken chorioallantoic membranes. α2β1 integrins were also found as targets of EMS16, rhinocetin, and vixapatin in HUVEC and HBMEC cells. These results also highlight the significance of antiangiogenesis as an anticancer mechanism of snake venom components (Dhananjaya and Sivashankari 2015). The affinity of the KTS motif to integrins was shown in several natural and synthesized peptides that showed nanomolar IC_50_ values towards the inhibition of adhesion of α1/α2 integrin to collagens (Momic et al. 2014). The involvement of the RGD tripeptide, the primary determinant of integrin binding, in the integrin-affinity is complex in reality. First, the affinity and selectivity of disintegrins for specific integrin subtypes is governed not by the RGD motif alone but by the flanking amino acids, the overall three-dimensional conformation constrained by disulfide bonding, and the dynamics of the integrin headpiece (Vasconcelos et al. 2021). RGD motifs alone are insufficient to predict function. On the other hand, the KTS, KGD, MGD, and MLD motifs, targeting various types of integrins, expand the pharmacological repertoire of disintegrins and complicate rational design (Vasconcelos et al. 2021). αvβ3 and αvβ5 promote angiogenesis and metastasis and the differences between αvβ3 and αvβ5 signaling pathways can specify how integrins confer distinct cellular behaviors (Vasconcelos et al. 2021).

Many disintegrins have been isolated from snake venoms, and their anticancer efficacies have been investigated. In many cases, their anticancer efficacies are linked to their properties to prevent cell adhesion and cell migration. DisBa01 is one of those integrins that was isolated from *Bothrops alternatus*. It is found to block αvβ3/VEGFR2 cross-talk in cancer cells and inhibit cell invasion, migration, and tubulogenesis. The molecules were concluded to be an anti-angiogenic substance (Danilucci et al. 2019). The characterization of DisBa01 as an inhibitor of αvβ3/VEGFR2 cross-talk represents mechanistic insight that moves beyond integrin blockade. However, the therapeutic efficacy of this finding needs to be proven since there are many endogenous and exogenous factors that can modulate αvβ3/VEGFR2 (Somanath et al. 2009). The efficacy of lebein at nanomolar concentrations (0.2 μM) against colon cancer cells is among the most promising reported for a disintegrin (Table 1), yet several critical caveats emerge upon deeper examination. The outcome that mojastin 1 and viridistatin 2 did not result in a synergistic effect may imply their targets are the same. On the other hand, they revealed similar IC_50_ values in BxPC-3 pancreatic cancer cells. In general, disintegrins have been well established to show strong antimetastatic and/or antiangiogenic effects by binding integrins. This property is crucial in tumor progression; however, their effective micromolar concentrations can hamper deeper research. Their combination with potential anticancer drugs that have reached the medical practice can lead to combined effects. Targeting integrins with venom-derived peptides offers a promising strategy for antimetastatic and anti-angiogenic cancer therapy.

Trigramin is a ~9 kDa cysteine-rich peptide, having a similar anticancer effect to triflavin, another cysteine-rich disintegrin that has a 7.5 kDa MW. These two disintegrins showed similar efficacies on MDA-MB-231 and MCF-7 breast cancer cells and PC-3 prostate cancer cells. At a 10 µg/mL, they reduced cell adhesion by ~80–85% compared to the control. Rhodostomin, another disintegrin, showed an even greater adhesion-reducing effect on those cells. In MDA-MB-231 and PC-3 cells, it reduced this capacity by ~90% (Yang et al. 2005). Lebetin 2, isolated from *Macrovipera lebetinus transmediterranea*, was found to bind α1β1 integrins, binding type I and type IV collagens. At a 500 µg/mL, it only halved the viability of PC12 pheochromocytoma cells for 72 h. Therefore, it can be claimed to be antitumorigenic and antiangiogenic (Morjen et al. 2018). These and other data discussed in this section show a wide range of anticancer potentials of various disintegrins. Targeting integrins with disintegrins led to greater efficacies. Therefore, in most cases, the terms ‘antimetastatic’ or ‘anti-angiogenic’ are used because of their greater efficacy in preventing cell migration and cell adhesion. Despite disintegrins being involved in various processes, binding to integrins deserves more attention as a general feature.

**Fig. 5:**
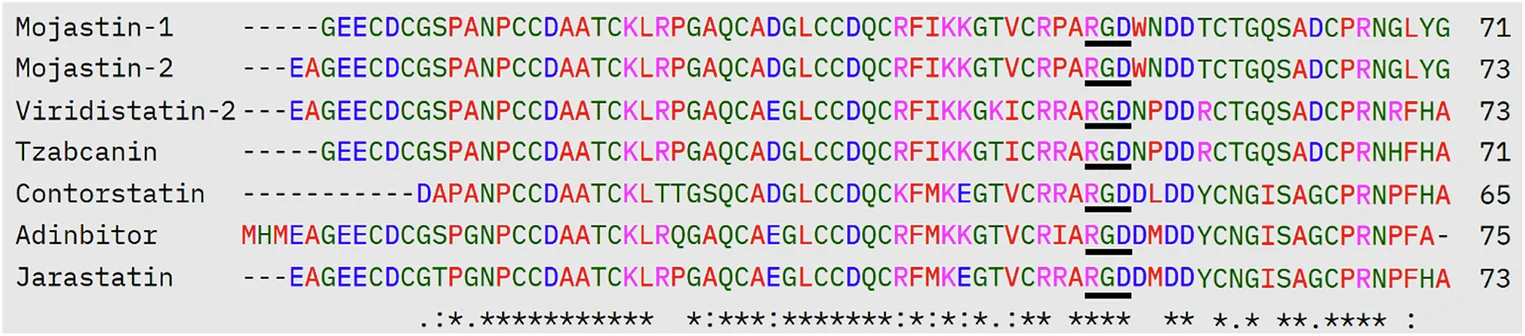
Amino acid sequence comparisons of medium-sized disintegrins

Disintegrins share a high degree of similarity in amino acid sequences. In Fig. 5, the comparisons of sequences of several disintegrins are shown. High similarity levels of pre-RGD motifs at the N-terminus can be noticed. We can observe almost the same sequences of mojastin-2, viridistatin-2, and tzabcain, differing only by a few amino acids. High similarity levels of primary structures enable us to predict similar anticancer potentials from these isoforms. Variances in their isoforms differing by their C-terminal sequences can cause varying cytotoxic potentials. RGDWN, RGDNP, RGDDL, and RGDDM motifs were found in these sequences. These effects likely arise from interactions beyond integrin binding, given the inconsistency with proliferation inhibition. Significant differences were not observed in cell migration inhibition levels resulting from these six mutants (Ramos et al. 2016).

The high sequence similarity among medium-sized disintegrins, particularly the near-identity of mojastin-2, viridistatin-2, and tzabcain, raises important questions about the utility of isolating and characterizing each variant individually. While minor variations in the C-terminal region and the specific RGD flanking motifs (RGDWN, RGDNP, RGDDL, and RGDDM) may imply influence on integrin selectivity, the functional consequences of these differences have not been systematically evaluated across a standardized panel of integrin subtypes using uniform assay conditions. This knowledge gap evidence suggests extensive functional redundancy. Furthermore, the presence of multiple isoforms within the same venom, sharing >90% sequence similarity, might also imply post-translational modifications. Without systematic structure-activity relationship studies that correlate specific amino acid differences with functional outcomes, the field risks investing considerable resources in characterizing numerous isoforms that might be functionally equivalent, thereby diverting attention from the more critical challenges of selectivity optimization, formulation, and delivery. These points can be specified to some extent by analysing the results gathered in Table 1, in which, we summarized the in vitro and in vivo outcomes of disintegrins. We need to mention that IC_50_ values from different sources may not be directly comparable because of some factors, including the differences in experimental design and cell models. Exposure time, assay condition, and developing conditions can be other factors affecting the comparisons.

The efficacy of lebein at nanomolar concentrations (0.2 µM) against colon cancer cells is among the most promising reported for a disintegrin (Table 1), yet several critical caveats emerge upon deeper examination. The outcome that mojastin 1 and viridistatin 2 did not result in a synergistic effect may imply their targets are the same. On the other hand, they revealed similar IC_50_ values in BxPC-3 pancreatic cancer cells. In general, disintegrins have been well established to show strong antimetastatic and/or antiangiogenic effects by binding integrins. This property is crucial in tumor progression; however, their effective micromolar concentrations can hamper deeper research. Their combination with potential anticancer drugs that have reached the medical practice can lead to combined effects. Targeting integrins with venom-derived peptides offers a promising strategy for antimetastatic and anti-angiogenic cancer therapy.

The disintegrin family of integrin-binding proteins has attracted interest as a source of antimetastatic and anti-angiogenic agents. The preclinical evidence reviewed in this section demonstrates their involvement in the disruption of cancer cell adhesion, migration, and invasion, and in some instances, can induce apoptosis and reduce tumor growth in xenograft models.

While integrin blockade represents a powerful antimetastatic strategy, snake venom also contains enzymes that attack cancer cells through entirely different mechanisms, including oxidative stress, membrane degradation, coagulation imbalance, etc. These diverse mechanisms offer the possibility of combination therapy, while multiple venom components target distinct vulnerabilities. In the next section, we discuss the enzymatic components of snake venom, revealing anticancer efficacies.

## Venomous enzymes

While disintegrins exert anticancer effects through non-enzymatic receptor blockade, a distinct and mechanistically more aggressive class of venom toxins operates through catalytic activity. Venomous enzymes, including snake venom metalloproteinases (SVMPs), phospholipases A2 (PLASs), L-amino acid oxidases (LAAOs), and snake venom serine proteases (SVSPs), represent a pharmacologically diverse arsenal that can directly damage cellular membranes, generate cytotoxic oxidative stress, disrupt mitochondrial bioenergetics, and interfere with coagulation cascades. The catalytic nature of these enzymes confers a fundamental advantage over receptor antagonists: enzyme molecules can influence multiple pathways (Babbi et al. 2020) and therefore can reveal higher cytotoxic potentials compared to molecules of non-catalytic mechanisms. In this section, we review the anticancer efficacies of venomous enzymes, paying particular attention to the selectivity of enzymatic cytotoxicity, the distinction between catalytic and non-catalytic mechanisms, the reproducibility of reported effects across independent studies, and the formidable challenges of translating into safe and effective therapeutics.

**Fig. 6:**
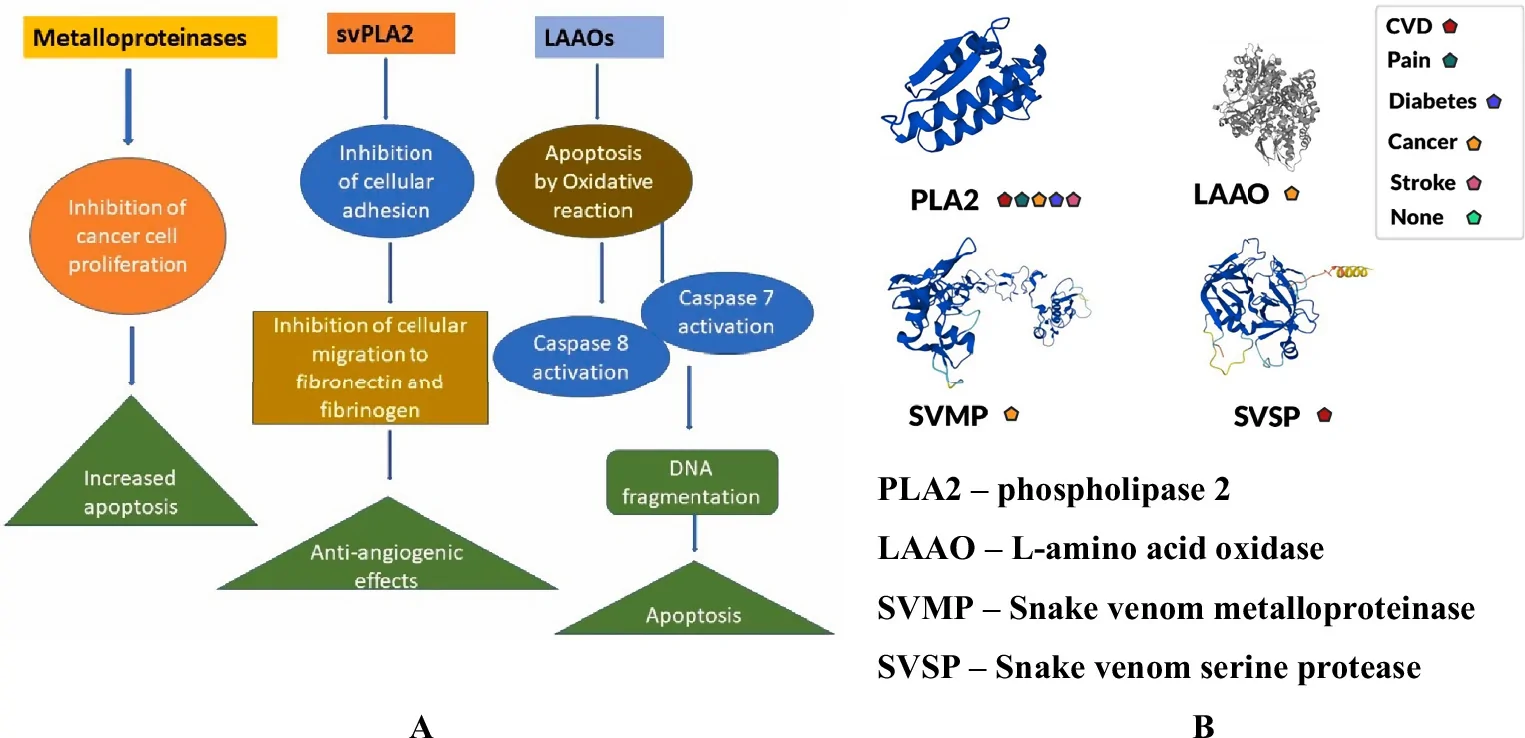
Venomous enzymes and their functions that cause anticancer efficacies. Figures **A** and **B** have been reused from (Akhtar et al. 2021) and (Bedraoui et al. 2024), respectively, with the permission of Elsevier

Enzymatic components, including metalloproteinases, phospholipases, amino acid oxidases, and serine proteases, significantly contribute to the antitumor properties of snake venom. Venom enzymes have a special role in the anticancer efficacy of the snake venom. The enzymes most investigated in snake venoms are metalloproteinases, phospholipases, L-amino acid oxidases (LAAO), and serine proteases. They are involved in various processes and, therefore, used in the therapy of various diseases (Fig. 6B). However, their anticancer roles have been well explained (Fig. 6A). Metalloproteinases inhibit cell proliferation, phospholipases inhibit cell adhesion and migration, LAAOs induce oxidative stress, and serine proteases induce an imbalance in blood coagulation. Several papers have been published on the roles of each of these enzymes in cancer therapy. These enzymes induce apoptosis and oxidative stress, highlighting their potential as antitumor agents despite challenges with effective dosing.

The classification of venom enzymes into metalloproteinases, phospholipases, L-amino acid oxidases, and serine proteases serves a useful organizational purpose; however, it is more complex in reality. These families are not mutually exclusive in their mechanisms. PLAs, for instance, can regulate oxidative stress (Yordanova et al. 2026), a function which is typically attributed to LAAOs (Costal-Oliveira et al. 2019), and on the other hand, LAAOs can trigger mitochondrial dysfunction (Yang et al. 2012), a function that typically belongs to metalloproteinases (Mohammad and Kowluru 2010). Physiological substrates of these enzymes in the context of cancer have been incompletely characterized. For example, while PLA2s catalyze the hydrolysis of membrane phospholipids, the specific lipid species targeted and the resulting bioactive lipid mediators (e.g., lysophospholipids, arachidonic acid derivatives) that mediate cytotoxicity have not been systematically profiled. Below in separate sections, we discussed the above-highlighted points of the venomous enzymes we mentioned.

### Metalloproteinases

Snake venom metalloproteinases (SVMPs) are a class of toxic proteins that disrupt cellular processes and exhibit cytotoxicity against cancer cells (Modahl et al. 2018). (Saranya et al. 2024) designed five anticancer peptides on the basis of SVMP. Based on molecular docking simulations, two of them, having arginine- and lysine-rich sequences, were predicted to lead to anticancer properties (Saranya et al. 2024). The high significance of charged amino acids in proteins and peptides has recently been demonstrated (Oliveira et al. 2022). Arginine-rich or lysine-rich peptides may exhibit enhanced delivery to tumor cells due to their potential interaction with albumin, which has a high surface density of aspartic and glutamic acids (Asrorov et al. 2024), and is often exploited for drug targeting (He et al. 2022). Since various substances in the venom lead to the occurrence of various mechanisms, establishing the mechanism of action of distinct molecules will further clarify their utilization potential as cytotoxic compounds. Pictolysine-III is a member of type III metalloproteinases that shows anticancer potential. It did not cause significant reductions in the cell viability levels of MDA-MB-231 breast cancer and RMF-621 fibroblast cells at 10 and 20 µg/mL. Significant reductions were observed at 50 µg/mL, with a greater effect on fibroblast cells. It was determined to be involved in mitochondrial bioenergetics. Calcium and magnesium ions increased the proteolytic activity, whereas zinc (Zn^2+^) and EDTA were found to lower the enzymic activity of the 62 kDa enzyme (Vivas-Ruiz et al. 2023). Further studies are required to elucidate their mechanisms and optimize therapeutic application.

### Phospholipases

Snake venom phospholipases are another large class of polypeptides revealing high cytotoxicity resulting from the inhibition of angiogenesis and cell migration. Their main function is to catalyze the isolation of fatty acids from phosphoglycerides. They are also found to be involved in the inhibition of angiogenesis and cell migration (Sobrinho et al. 2016). (Santos et al. 2024) report the inhibition of the cell growth and metastasis of F549 lung cancer by a phospholipase, termed MjTX-II PLA2, derived from *Bothrops moojeni*. Its 20 µg resulted in 2.5-fold suppression of A549 cell migration. The protein holding 112 amino acids revealed antiangiogenesis activity in vitro and ex vivo. The protein was claimed to be selective for cancer cells over noncancerous lung cells (Santos et al. 2024). Crotoxins are types of basic phospholipases found in snake venoms. A 121 amino acid-long isoform of crotoxin isolated from the species of the *Crotalus* genus was found to be toxic to cancer cells but not to noncancerous cells. The IC_50_ values of the protein in MDA-MB-231 and MCF-7 cells were 2.95 and 2.31 µg/mL for 24 h, respectively. Concentration-dependent changes were observed in MCF10A, HMEC, and HDFa noncancerous cells following the treatment at 0.2–1.0 µg/mL, but these alterations were insignificant (Bezerra and Motti 2024). (Almeida et al. 2021) report that a 50 µM crotoxin almost halved the viability levels of MCF-7aro, aromatase inhibitor-resistant breast cancer cells, whereas no significant changes were observed in HFF-1 human foreskin fibroblasts (Almeida et al. 2021). These results explain the specificity of the protein to cancer cells. A phospholipase BthTX-I from the venom was another molecule possessing an antitumor effect; it inhibited S180 solid tumors in mice. The cytotoxicity percentages of the 1 µg/mL phospholipase on MPC, Jurkat, SKBR3, B16F10, and S180 cells were 80, 90, 40, 80, and 75%, respectively (Gebrim et al. 2009). Their selective toxicity towards cancer cells supports their potential in targeted cancer therapy.

PLA2s represent one of the most intensively studied classes of venom enzymes, and the evidence for their anticancer activity, particularly their ability to inhibit angiogenesis and cell migration, is among the most compelling in the venom field. The above-discussed data show IC_50_ values ranging between 0.1 and ~3 µg/mL in several cases. The mechanisms underlying PLS2 cytotoxicity remain incompletely defined, while the inhibition of angiogenesis and cell migration is well documented, it is not completely clear whether these effects result from enzymatic catalysis (e.g., phospholipid hydrolysis generating bioactive lipid mediators) (Santos et al. 2024) or from non-enzymatic interactions (e.g., binding to specific receptors such as the PLA2 receptor) (Bernard and Vindrieux 2014). This distinction is critical because non-enzymatic mechanisms would be amenable to engineering catalytically inactive variants with reduced systemic toxicity, whereas enzymatic mechanisms, by their very nature, are more complicate to control spatially and temporally.

### L-amino acid oxidases

**Fig. 7:**
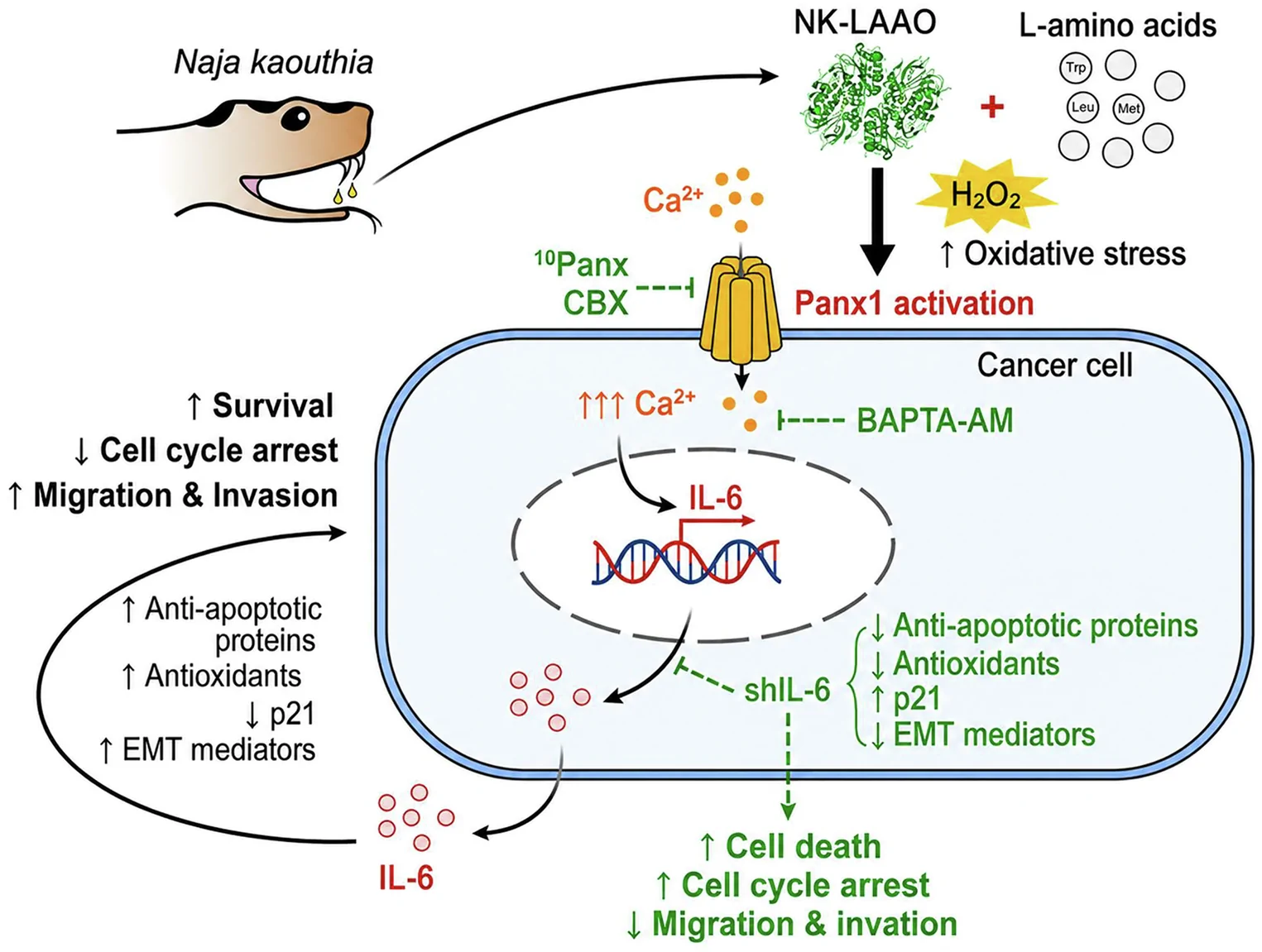
Acting mechanisms of LAAO leading to cytotoxic effects. The figure has been reused from (Truong et al. 2023) with the permission of Elsevier

L-amino acid oxidases (LAAOs) are highly cytotoxic enzymes due to their potential to induce oxidative stress and apoptosis, leading to the induction of cell death and cell cycle arrest (Fig. 7). A 0.3 µM LAAO isolated from *Naja kaouthia* was shown to induce caspase-3 and the expression of p21 RNA. Its 0.1 µM caused significant changes in IL-1β, IL-6, and TNF-α levels. The protein was found to induce oxidative stress both outside and inside the cell. The authors claimed the significant role of substrate composition rather than glycans on the enzyme surface (Truong et al. 2023).

Rusvinoxidase, a type of ~57 kDa LAAO isolated from *Daboia russelii russelii*, was found to have high cytotoxicity against MCF-7 breast cancer cells. Its specific activity was 3.6 times greater than that of viper venom. Its concentration-dependent cytotoxic activity in 24 h was significantly higher than that of the anticancer drug cytosine-β-D-arabinofuranoside, a selective DNA synthesis inhibitor. Its IC_50_ (83 nM) value was 3.6 times greater than that of viper venom. The same dosage resulted in mild inhibition of human HEK cells, indicating the low cytotoxicity against normal cells. Furthermore, rusvinoxidase concentration- and time-dependently decreased the mitochondrial transmembrane potential of MCF-7 cells and threefold increased ROS generation compared to the control group. Following the treatment, caspase-8, –9, and –7 levels increased significantly in 24 h. The LAAO enzymatic activity was accompanied by the induction of ROS and caspase-7 to reveal the antitumor potential (Mukherjee et al. 2015). LAAO isolated from *Calloselasma rhodostoma* showed immunotherapeutic potential. It induced the release of IL-6, IL-1β, and LTB4. A 1.5 µg/mL of LAAO increased the amount of IL-6 and IL-1β in HL-60 cells by >2.5-fold. In HepG2 cells, similar changes were observed in IL-1β quantity by a 1.5 µg/mL drug. Oxidative stress, combined with caspase activation, was claimed to lead to apoptosis (Costa et al. 2017). In another work, the cytotoxicity of L-amino acid oxidase, isolated from *O. hannah*, was studied in colorectal cancer, stomach cancer, and murine melanoma cells. The mechanism of action was linked with thymidine incorporation (Ahn et al. 1997). Cytotoxic effects of L-amino acid oxidases were observed in the range of 1–100 µg/mL. The highest effects were observed in A549 (0.3–3.0 µg/mL), MKN-45 and RKO (0.1–20 µg/mL), and HeLa and K562 (2.5–10 µg/mL) cells, respectively (Izidoro et al. 2014).

LAAOs were reported to reveal oxidative stress and caspase activation leading to apoptosis. Their µg/mL effective doses may not make them attractive for cancer therapy. Since cancer cells exhibit heightened sensitivity to oxidative stress due to their elevated ROS levels and impaired antioxidative defense system (Snezhkina et al. 2019), the induction of exogenous oxidative stress through enzymatic H2O2 generation may not be sufficient to control tumor tissues. Animal experiments are required to clarify the efficacy of LAAOs since predicting enzyme in vivo activity based on in vitro results can be misleading due to assays using saturated substrates (Truong et al. 2023). Deeper analyses of the enzyme immunostimulatory effects can clarify the main pathways causing anticancer efficacy of the enzyme. Moreover, comparative analyses of the enzyme from different sources can also serve to understand the enzyme capacity more profoundly.

### Serine proteases

Serine proteases or proteinases are a group of snake venom proteins that interfere with coagulation and cellular signaling. Their functions are involved in the blood coagulation cascade, causing an imbalance in the hemostatic system. They cleave peptide bonds around Arg or Lys at specific positions (Serrano 2013). Batroxobin is one of the serine protease enzymes isolated from *Bothrops moojeni*. Its combination with NK cells effectively inhibited the tumor size of Lewis lung cancer; compared to the control, the treatment halved the tumor size, which was twice as effective as their separate effects. Both batroxobin and NK cells significantly reduced MMP2 and MMP9 matrix metalloproteinases, and their combination resulted in synergistic effects (Liu et al. 2024). Batroxobin possesses high N-end similarity (VIGGDECDINEHPFL) to other polypeptides with the VIGGDECNINEHRFL sequences found in enzymes termed AHP-Ka, Crotalase, and Ancrod (Zhang et al. 2012). One serine protease, termed Kn-Ba, was reported to have fibrinogenolytic and kinin-releasing activities (Megale Â et al. 2018). Another serine protease having a 28 kDa MW isolated from *Bothrops jararaca* was reported to show fibrinogenolytic activity. It made one-thirtieth part of the crude venom, and the purification resulted in threefold higher esterase activity (Carone et al. 2018). The main problem of serine proteases is their limited doses in original sources, and therefore, they have started to be obtained recombinantly (Boldrini-França et al. 2019). Collinein-1 is another serine protease, the cytotoxic potential of which was studied in three cell types. At 2, 4, and 8 µM concentrations, it significantly reduced the MCF7 cell viability concentration-dependently, but not in MCF10 non-malignant breast epithelial cells. Besides, significant differences were not observed in HepG2 human liver cancer cells. Moreover, the protein was found to have potassium channel-blocking activity (Boldrini-França et al. 2020).

Serine proteases occupy a unique niche among venom enzymes in that their primary mechanism of action, interference with the coagulation cascade, is fundamentally different from the direct cytotoxicity exhibited by PLA2s and LAAOs. This distinction has important implications for their evaluation as anticancer agents. The therapeutic rationale for serine proteases in oncology is less direct than for other venom enzymes; rather than killing cancer cells directly, serine proteases are considered to exert antitumor effects by modulating tumor microenvironment and altering immune cell infiltration (Tagirasa and Yoo 2022). Validation and quantification of this indirect action is a complex question. The major limitation of this class of enzymes to translation is their minor quantity in venoms. Comparative study of known serine proteases for their anticancer efficacy by obtaining recombinant forms in preparative amounts can enable clarification and specification to open a door to in vivo experiments.

Cytotoxic enzymes of snake venom origin, including metalloproteinases, LAAOs, PLA2, and serine proteases, show anticancer potential resulting from the formation of oxidative stress, apoptosis, and cell cycle stress. However, their micromolar effective concentrations may prevent deeper research. Therefore, some alternative approaches, such as combining the individual proteins with other anticancer agents, can lead to their enhanced anticancer potential. Recombinant production and combination strategies may enhance their therapeutic efficacy.

## Three-finger toxins (3FTx)

Three-finger toxins (3FTx) constitute one of the most structurally and functionally diverse families of snake venom proteins, characterized by a conserved three-fingered β-sheet fold stabilized by four to five disulfide bridges. This scaffold, having exceptional structural stability, targets nicotinic acetylcholine receptors (ligand-gated ion channels) (Shenkarev et al. 2022), muscarinic receptors (Kessler et al. 2017), and membrane phospholipids (Dubovskii et al. 2005). Within this family, cytotoxins and cardiotoxins attracted particular interest for their potent and, in some cases, selective anticancer activity. Unlike the enzymatic toxins discussed in the previous section, 3FTxs exert cytotoxicity primarily through membrane disruption and the activation of intracellular apoptotic cascades. Their relatively small size, consisting of 60–80 amino acid residues (Utkin 2019), makes them attractive candidates for polypeptide-based drug development. Most researched members, called cytotoxins and cardiotoxins, are known to reveal anticancer potential (Guo et al. 2024). However, the mechanistic distinction between membrane disruption (which is inherently non-specific) and receptor-mediated apoptosis remains unknown, which determines the therapeutic prospects of this class. In this section, we discuss the anticancer efficacy of some members of 3FTxs belonging to cytotoxins and cardiotoxins.

### Cytotoxins

Cytotoxins from cobra venoms show high activity against a range of cancer cell types. Cytotoxins isolated from *Naja* species have been established to be effective against several cancer cells at micromolar concentrations (Boughanmi et al. 2024). Cytotoxin 2a (*N. sumatrana*), cytotoxin 3 (*N. kaouthia*), and cytotoxin 32 (*N. naja*) are well-known members that showed good anticancer effects on MCF-7 cancer cells with IC_50_ values of 9.1, 12.23, and 2.5 µg/mL, respectively. They showed greater effects on A549 lung cancer cells: cytotoxin 3 (*N. kaouthia*) and cytotoxin 2a (*N. sumatrana*) revealed IC_50_ values of 1.22 and 0.88 µg/mL in A549 cells, respectively. The IC_50_ values of cytotoxin 3 (*N. kaouthia*) and cytotoxin 2a (*N. sumatrana*) in PC-3 prostate cancer cells were 4.46 and 3.13 µg/mL, respectively (Chong et al. 2020). Similar IC_50_ levels of other isoforms were reported in HL-60 leukemia cells (Feofanov et al. 2004). Five isoforms isolated from *N. anchietae* and *N. senegalensis*, termed CTNanc1, CTNanc2, CTNanc3, CTNsen1, and CTNsen3, showed high or moderate IC_50_ values in U87 glioblastoma cells, ranging from 5.33 to 54.69 µg/mL. However, they showed very low cytotoxicity in U251 or T98G cells; their IC_50_ values ranged from 390 to 476 µg/mL or were found ineffective (Boughanmi et al. 2024). Proteomic analyses integrated with *in silico* studies may resolve these order-of-magnitude discrepancies.

Cytotoxin II, a toxic polypeptide with a MW ~ 17.5 kDa (Aderka et al. 1985), was found to have a greater anticancer effect than paclitaxel, with an eight-fold lower IC_50_ value in MCF-7 breast cancer cells. The activation of caspase was suggested as one of the probable causes leading to apoptosis (Ebrahim et al. 2014). The cell inhibition and induction of apoptosis in cancer cells by snake venom were also linked with increased Ca^2+^ influx, enhancement of cytochrome C release, and altered levels of proteins controlling the cell cycle (Gasanov et al. 2014). (Derakhshani et al. 2020) reported the cytotoxic effect of a ~ 6.6 kDa MW recombinant cytotoxin II. Its IC_50_ values in SK-MEL-3 and HFF-2 cells were 17.7 and 24.86 µg/mL, respectively. Its 17.7 µg/mL concentration induced caspase-9 and –8 RNA expression levels 3.5-fold and by ~60%. The expressions of miR-214 and MMP-3 were downregulated. Two regions of the protein were predicted as B-cell epitopes (Derakhshani et al. 2020).

Cytotoxin 10 isolated from *N. kaouthia* was determined as another effective anticancer polypeptide against a wide spectrum of cancer cells. In four breast and lung cancer types, it showed very similar IC_50_ values ranging from 6.04 to 8.71 µg/mL, which was twice lower than that in HEK-293T immortalized kidney cells. The calculated IC_50_ values in MDA-MB-231, A549, and HCI-H522 cells were 2–4 times smaller than those of crude snake venom. A 9 µg/mL concentration increased the levels of the Bax/Bcl-2 ratio and cleaved PARP several-fold in MCF-7 and A549 cells (Basumatary et al. 2024).

Comparative analysis of cytotoxin-1N and cytotoxin-2N in three adenocarcinoma cells showed their similar effects. Both toxins at 3 µM amounts almost completely reduced the cell viability of A549 non-small cell lung adenocarcinoma cells. Their 10 µM concentrations reduced the viability of MDA-MB-231 breast adenocarcinoma cells by <30 and <15%, respectively. At 30 µM concentrations, cytotoxin-1N and cytotoxin-2N reduced the viability level of HT-29 colorectal adenocarcinoma cells to <15 and <40%. The lowest potential belonged to cytotoxin-3N; a 30 µM concentration did not cause remarkable effects on MDA-MB-231 and HT-29 cells. The effects of cytotoxin-4N looked similar to cytotoxin-1N and -2N. It showed high potential on A549 cells, an average impact on MDA-MB-231, and a low influence on HT-29 cells (Conlon et al. 2020).

Nubein is another anticancer peptide isolated from the *Naja nubiae* cobra. The peptide with a MW of 6.8 kDa was characterized by the LKCNQL sequence. Other isoforms were found to be 60 amino acids in length and hold 9 lysine and two arginine residues, making them basic polypeptides. It reduced the A375 and A2780 cancer cells more effectively than the PNT2 normal cells. A ~ 1 µM concentration reduced cancer cell viability by 25% while reducing noncancerous cell viability by ~50%. The peptide was found to be internalized in cancer cells and cell organelles (Abdel-Ghani et al. 2019). In summary, the mechanisms of action of cytotoxins include caspase activation and mitochondrial disruption, meriting further development. The authors’ determination that the peptide is internalized raises an interesting question about the internal activity of cytotoxins other than caspase-related pathways.

The evidence for cytotoxic activity of snake venom cytotoxins across multiple cancer cell lines is extensive and clear. However, several points might prevent their translational significance. The above-discussed data show their high micromolar IC_50_ values (greater than 20–30 µM) that make them less attractive in terms of their anticancer efficacy. These high doses complicate the prediction of their actions, whether involved in specific membrane disruption or other nonspecific actions that might result from their internalization in cancer cells. Their utilization with lower molecular anticancer drugs in combination can clarify further development in this discipline. Establishing the expression levels of their recombinant forms is another question to improve their cost-efficacy.

### Cardiotoxins

Cardiotoxins, while primarily cardiotoxic, also demonstrate significant anticancer properties. Cardiotoxins, a group of cytotoxins mainly damaging cardiomyocytes, have also been paid attention to as anticancer means. In one work, a cardiotoxin termed CTX3 was found to reduce the viability of U937 leukemia cells via the degradation of the catalytic subunit of protein phosphatase in a Ca^2+^-dependent manner, leading to autophagy and apoptosis (Chiou et al. 2019). The occurrence of apoptosis of HL-60 human leukemia cells was linked with the induction of caspase-9, –12, cytochrome C, Bax/Bcl-2, and others. The toxin was concluded to target several sites of apoptotic pathways (Chien et al. 2008). The induction of caspase-3 and -9 and cytochrome C release resulted from the treatment of SK-N-SH neuroblastoma cells with CTX-3. Besides, apoptosis was linked with the enhanced levels of ROS and mitochondrial permeability transition (Chen et al. 2008). Another work showed high anticancer efficacies in three oral cancer cells. Its 0.5 µM concentration resulted in <60, <40, and <20% viability levels of SAS, CAL-27, and Ca9-22 oral cancer cells, respectively. The toxic potential of the polypeptide was attributed to apoptosis and cell cycle arrest at the S-phase (Chien et al. 2010). The activation of caspase-9 and –3 was concluded to lead to apoptosis in Ca9-22 cells (Chien et al. 2009).

The induction of Bax and Bad, caspase-3, –9, and cytochrome C resulted from the treatment of MDA-MB-231 cells with CTX3, leading to apoptosis. A 0.15 µM concentration of CTX3 lowered the cell viability to <40% for 18 h (Lin et al. 2010). The effect of CTX3 on MDA-MB-231 breast cancer cells at higher 1 and 2 µM concentrations did not result in significant reductions in the cell viability levels. These concentrations significantly reduced cell migration and cell invasion levels. Seemingly, the suppression in phosphorylation levels of ERK1/2 and PI3K/AKT and the reduction in MMP-9 activity could not induce toxic effects at these concentrations (Tsai et al. 2013). A 2 µM concentration of the protein reduced the cell viability level of MCF-7 breast cancer cells to 60%, with no significant differences in the effects of the toxin on MCF-7 and HEK-293 cells. A 5 µM concentration of the protein reduced this level to <40% (Chiu et al. 2009). CTX3 also showed similar anticancer efficacy in HepG2 cells, as 6.25 µM of the toxin lowered the cell viability to ~25% in 24 h. A similar mechanism was concluded to lead to protein anticancer activity, including cell cycle arrest at S-phase and caspase-dependent apoptosis (Chen et al. 2009).

Cytotoxins and cardiotoxins demonstrated selective toxicity towards cancer cells by triggering caspase-dependent pathways. Their ability to induce apoptosis in cancer cells supports their investigation as anticancer agents. However, they were also found to possess micromolar effectiveness that might prevent deeper research. Therefore, the approaches needed for other venom toxins may be applied to their further development.

Cardiotoxins present a challenging case for therapeutic development, as their names signal a significant safety liability. Although they have been mechanistically well established, their translational relevance is hampered by their micromolar efficacy against cancer cells. The main mechanism that makes cardiotoxins cytotoxic to cancer cells – membrane disruption and induction of apoptosis – is the same mechanism that causes their cardiotoxicity, and there is no evidence that cancer cells are more sensitive to cardiotoxins than cardiomyocytes. Therefore, further research is needed to study their comparative effects on cancer cells and cardiomyocytes as well as normal cells. In this term, in vivo experiments in mice might clarify these points.

Structural homology between cytotoxins and cardiotoxins, differing from each other with a few amino acids in several places (Konshina et al. 2012), raises a question about the structure-activity relationship. Without systematic structure-activity relationship studies that correlate specific loop sequences with specific biological activities, the field risks attributing therapeutic significance to structural differences that may be pharmacologically irrelevant.

## Kunitz-type serine protease inhibitors (KSPIs)

Kunitz-type serine protease inhibitors (KTSP) represent one of the most structurally conserved yet functionally diverse families of snake venom proteins. Characterized by a canonical Kunitz fold, consisted of approximately 50–70 amino acids stabilized by three conserved disulfide bridges, these molecules inhibit a broad spectrum of serine proteases, including trypsin, chymotrypsin, plasmin, thrombin, and coagulation factors Xa and Xia (García-Fernández et al. 2016). Dual functionality that includes protease inhibition and ion channel modulation make them different from other venom components that may be particularly relevant to cancer therapy (García-Fernández et al. 2016). Differing from membrane-disrupting 3FTxs or catalytically active enzymes discussed in previous sections, KSPIs exert their biological effects through high-affinity to molecular targets, offering selectivity (Ranasinghe and McManus 2013), can be an effective option for protein engineering. In this section, we critically evaluate the evidence for anticancer activity among venom-derived KSPIs, paying particular attention to the selectivity of their protease-inhibitory and ion-channel blocking activities.

Serine proteases play significant roles in many biological functions. They are involved in coagulation, inflammation, and other important processes that deserve special attention (Almonte and Sweatt 2011). KSPI inhibit protease activity and exhibit anti-angiogenic and antimetastatic effects. Researchers have recently focused on these compounds due to their putative potential in cancer therapy. Snake venom contains various KSPIs that could be used as an anticancer agent. In one work, a KSPI molecule, termed flavikunin and derived from *Bungarus flaviceps*, was reported to inhibit several proteases, with the highest effect on plasmin. One amino acid-substituted recombinant form of flavikunin increased plasmin-inhibiting and trypsin-inhibiting activities slightly, whereas the thrombin-inhibiting effect was reduced several-fold (Kaur et al. 2019). These results explain the significance of proteomics and obtaining recombinant forms. The engineering of flavikunin variants through a single amino acid substitution represents a sophisticated approach to optimizing protease specificity and represents the type of structure-activity relationship study that is solely needed across the venom field. The alterations in plasmin-, trypsin-, and thrombin-inhibiting activities are mechanistically informative and provide a proof-of-principle KSPI selectivity can be rationally modulated. A peptide named PEP2 is another inhibitor of proteases. The polypeptide isolated from a fraction with <10 kDa MW inhibited cysteine and serine proteases. The peptide was found to effectively inhibit cathepsin, a cysteine protease inhibitor, and elastase-1, the serine protease (Kodama et al. 2020). The isolation and characterization of PEP2 as a dual inhibitor of cysteine and serine proteases is significant since cathepsin is overexpressed in cancer and involved in tumor invasion and metastasis (Zhang et al. 2014, Fujii et al. 2025).

**Fig. 8:**
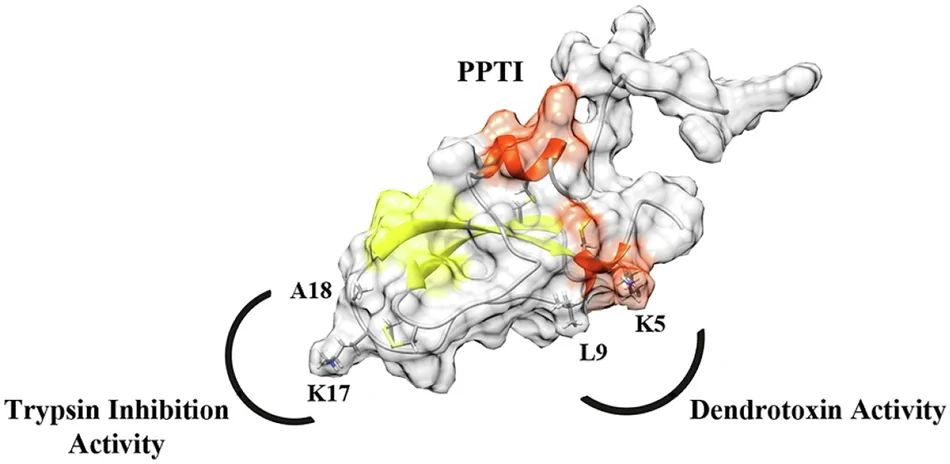
A 3D structure of PPTI and its trypsin-inhibiting and dendrotoxin activity sites. The figure has been reused from (Banijamali et al. 2019) with the permission of Elsevier

*Pseudocerastes persicus* trypsin inhibitor, abbreviated as PPTI, is another active polypeptide. A ~ 7.6 kDa protein consisting of 68 amino acids revealed trypsin-inhibiting and dendrotoxin activity (Fig. 8). A 200 nM protein competitively inhibited trypsin; that was a similar level compared to other protease inhibitors (Banijamali et al. 2019).

The characterization of PPTI as a dual-function molecule with both trypsin-inhibiting and dendrotoxin activity exemplifies the functional complexity of KSPIs and the challenges of understanding their mechanism of action. The three-dimensional structure of PPTI shows distinct responsible sites for trypsin and dendrotoxin activity, which explains that these two activities of the protein are independent. Based on correlations between trypsin inhibitors and cancer development (Paju et al. 2007, Gaber et al. 2009), some anticancer features can not be predicted for PPTI. A deeper research can clarify its cytotoxic potential against cancer cells.

**Fig. 9:**
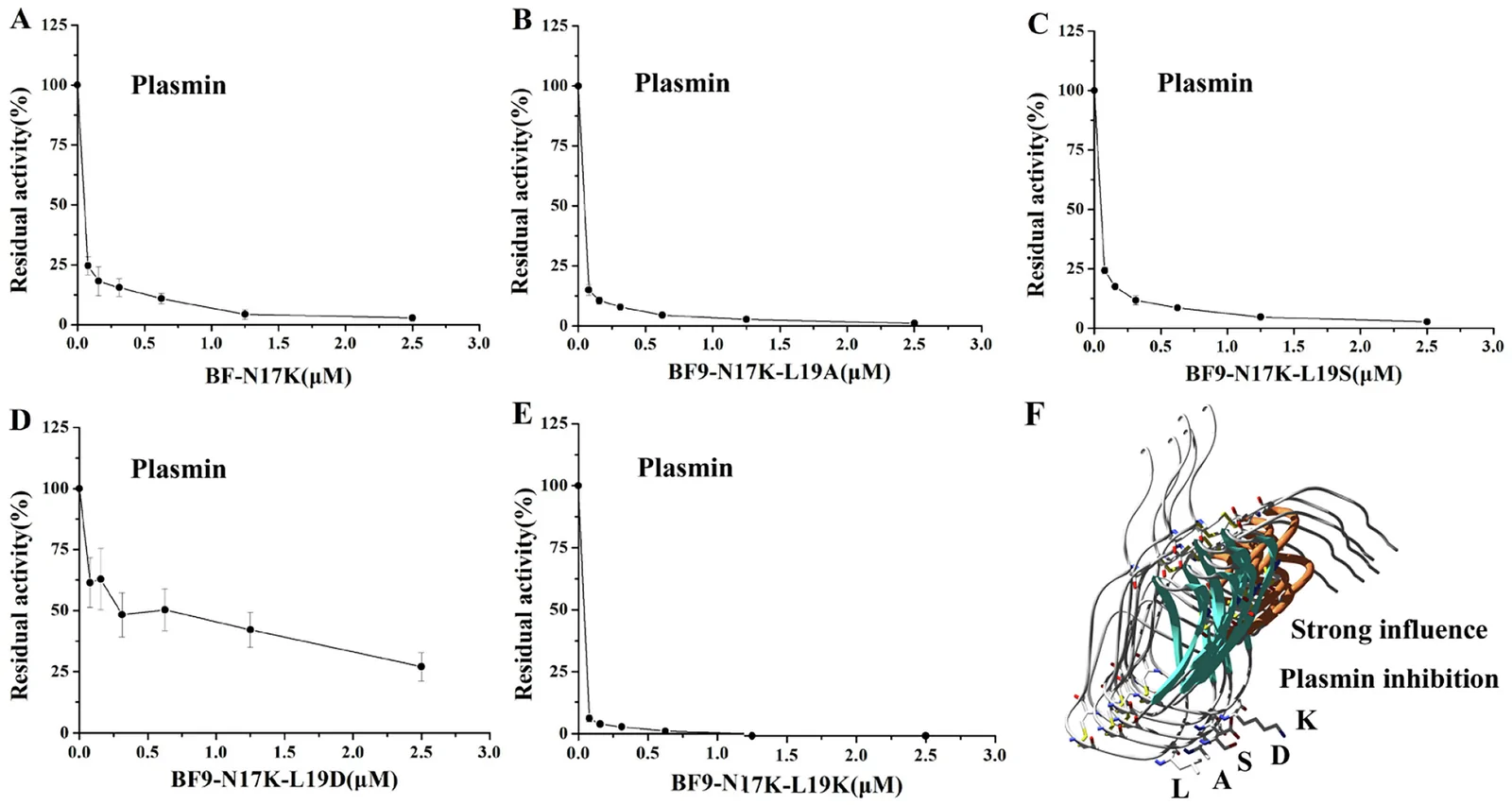
Inhibitory effects of BF9-N17K and L19-substituted isoforms towards serine protease plasmin. **A**–**E** indicate the residual activity of BF-N17K, BF9-N17-L19A, BF9﻿-N17-L19S, BF9﻿-N17-L19D, and BF9-N17K-L19K. F shows comparative analyses of *-L19X-substituted proteins in ribbon-like structure. The figure has been reused from (Ding et al. 2025) with the permission of MDPI

Another KSPI, termed PIVL and derived from *Macrovipera lebetina transmediterranea*, was recombinantly obtained in *E. coli*. Neither a 1 µM natural nor a recombinant form caused significant alterations in the viability levels of U87 glioblastoma cells for 24 and 48 h. Significant reductions were observed in the cell adhesion levels. A 10 nM concentration resulted in significant reductions in cell migration and invasion levels; a 100 nM concentration lowered these levels by ~40% and ~50%, respectively. Their 1 µM concentration halved the total vessel length studied on the CAM model. These results explain the similar efficacies of these inhibitors as disintegrins, preventing angiogenesis (Morjen et al. 2022). The antiangiogenic property of PIVL was found to be mediated by its RGD-like motif. A 100 nM concentration was also found to halve tube formation, showing its high antiangiogenic activity (Morjen et al. 2014). A 7691.7 Da isoform of PIVL, consisting of 67 amino acid residues, was found to be cytotoxic to human glioblastoma cells. Its 100 nM concentration halved the migration of U87 cells and reduced the cell invasion level by over 60%. The RGN motif was found to be responsible for the formation of the anticancer effect (Morjen et al. 2013). The characterization of PIVL as a KSPI with anti-angiogenic and anti-metastatic effects represents one of the most comprehensive investigations of a venom-derived KSPI in the cancer context. The observation that PIVL reduces U87 glioblastoma cell migration and invasion at concentrations as low as 10 nM, and halts tube formation at 100 nM, suggests a level of potency that approaches anti-angiogenic agents. The finding that the RGN motif, rather than the Kunitz loop, is responsible for the anti-angiogenic activity of PIVL is noteworthy. It suggests a mechanism of action that is independent of protease inhibition and may be mediated through direct integrin binding. Another snake toxin, termed BF9, inhibited α-chymotrypsin and potassium ion channels. Its ion channel-inhibiting IC_50_ value was 120 nM (Yang et al. 2014). These results suggest the presence of numerous active sites of snake venom proteins that can lead to various activities. In one unique work, the N17 amino acid in the composition of BF9 was substituted with other amino acids (Tyr, Y; Asp, D; Gly, G; His, H; Leu, L; Lys, K; Arg, R; Thr, T) to enhance chymotrypsin-inhibiting activity. Among nine one-amino acid residue-differing isoforms, the BF9-N17Y (Asp→Tyr) isoform demonstrated the highest enzyme-inhibiting activity. Four other isoforms – BF9-N17H, BF9-N17L, BF9-N17K, and BF9-N17R also enhanced the inhibitory rate by ~20% compared to BF19. BF9-N17K, BF9-N17H, and BF9-N17R were concluded as three coagulation pathway-inhibiting isoforms (Ding et al. 2018). Further development was carried out by changing Leu at position 19 (L19) with other amino acids (Fig. 9). Leucine substitution with aspartic acid, BF9-N17K-L19D (Fig. 9D), resulted in reverse effects compared to BF9-N17K (Fig. 9A). The isoform BF9-N17K-L19-K enhanced the trypsin-inhibiting efficacy several times (Fig. 9E). Significant differences among the isoforms were not observed in serine protease Xa- and IIa-inhibiting activities. Moreover, these substitutions resulted in controversial results in plasmin- and serine protease XIa-inhibiting efficacies (Ding et al. 2025). These results show the significance of researching proteomic analyses and engineering various isoforms.

The systematic engineering of BF9 variants shows the efficacy of the approach to improve the specificity of the engineered proteins for selection. In the above work, despite one of the isoforms revealing the improved enzyme-inhibiting activity, it does not claim in vitro efficacy. Greater in vitro efficacy may even belong to other forms due to the presence and expression levels of several isoforms. Even more diverse outcomes can be expected in vivo. Therefore, the continuation of the effects of various isoforms in vitro and in vivo can clarify and specify the effectiveness of some isoforms.

KSPIs are probably the most potent anticancer agents among the proteins and peptides isolated from the snake venoms. They often showed nanomolar effectiveness in several cancer cells. However, their translational relevance is complicated by some factors that are underappreciated in the literature. First, the protease-inhibitory activity of KSPIs is competitive and stoichiometric, so sustained inhibition in vivo requires inhibitor concentration above target protease levels, which may be difficult in the protease-rich tumor microenvironment. Secondly, the dual functionality of many KSPIs, including protease inhibition and ion channel blockage, might cause a complex pharmacological profile to identify the responsive function. Further development in this discipline requires systematic approaches to improve the efficacy of some potential isoforms that can result from the amino acid substitution that was shown in specific cases.

KSPIs represent a structurally elegant and functionally diverse class of snake venom proteins with demonstrated anti-angiogenic, anti-metastatic, and, in some cases, direct cytotoxic activity against cancer cells. KSPI can attract attention as an effective template for protein engineering and structure-based drug design. The above-discussed evidence in this section suggests that KSPIs may exhibit anticancer effects through at lest three mechanisms: direct inhibition of serine proteases involved in cancer progression (e.g., plasmin, thrombin) (1), disintegrin-like interactions mediated by non-canonical motifs (e.g., RGN) (2), and modulation of ion channel activity with potential consequences for cancer cell proliferation and survival.

## Lower molecular weight peptides

The transition from large, structurally complex venom proteins to lower molecular weight peptides represents a strategic pivot in the development of venom-derived anticancer agents. Differing from the above-discussed disintegrins, venomous enzymes, 3FTxs, and KSPIs, low molecular weight peptides, typically defined as those with <5 kDa MW, offer several distinct advantages for therapeutic development. In terms of anticancer agents, their putative lower immunogenicity due to their smaller size can be advantageous compared to larger proteins. By delivery aspects, they might face fewer barriers than larger proteins to reach the tumor. On the other hand, these advantages are counterbalanced by equally significant limitations: small peptides are more easily subjected to proteolytic degradation, they might reveal reduced target affinity compared to larger proteins, and they might lack high specificity for molecular recognition. The lower molecular weight peptides of snake venom origin, including cathelicidins, crotalicidin, and various synthetic derivatives containing integrin-binding motifs (e.g., KTS), represent a diverse class of molecules that exploit membrane disruption, integrin antagonism, and metabolic modulation to exert anticancer effects. In this section, we discuss anticancer efficacies of small peptides, paying particular attention to their mechanism of action, selectivity, features related to their sizes and charges, and translational relevance of their in vitro and in vivo results.

Lower molecular weight peptides deserve special attention as anticancer means. Due to the ease of synthesis and cost-effectiveness of peptides compared to proteins, they have received significant research interest. (Naamneh et al. 2024) obtained several peptides containing the KTS sequence – W-***KTS***-R-TSHY-TGKSDG modified with meta-aminobenzoic acid (mAba). Among seven peptides, ***KTS***-**W**-***KTS-*mAba**-R-TSHY-TGKSDG and W-***KTS***-**mAba**-**R**-**mAba**-R-TSHY-TGKSDG showed 442 and 324 µM IC_50_ values in the α1β1-binding in vitro assay, respectively, that were at least twice lower than that of their precursor (970 µM). A 10 mg/kg administration of W-***KTS***-**mAba**-**R**-**mAba**-R-TSHY-TGKSDG peptide very effectively inhibited the tumor growth in mice bearing B16 melanoma, which was a several-fold lower volume compared to the control. Besides, it extended the total animal survival rate by more than 40 days, which was very similar to that of healthy animals for over 70 days. The authors found the most effective anticancer compound among several variants. Molecular modeling showed the affinity between the binding site of integrin and the synthetic peptide with high anticancer potential (Coutinho et al. 2023). Another peptide, having a sequence ENWPHPQIPP, termed Bj-PRO-10c, which originated from the venom of *Bothrops jararaca*, was found effective against cancer cells. A 100 nM concentration of the peptide significantly reduced the proliferation rate of SH-SY5Y neuroblastoma cells for 24 h and halved that level for the next 48 h. The peptide, an activator of argininosuccinate synthase, was concluded to be a potential anticancer and antimetastasis agent (Coutinho et al. 2023).

Lysine-rich peptides deserve special attention among lower MW peptides of snake venom origin. (Falcao et al. 2015) reported the effects of crotalicidin (KRFKKFFKKVKKSVKKRLKKIFKKPMVIGVTIPF, Ctn) on several cancer and noncancerous cells compared to its partial sequences KRFKKFFKKVKKSV (Ctn_1-14_) and KKRLKKIFKKPMVIGVTIPF (Ctn_15-34_). Compared to the partial sequences, the full sequence showed several-fold higher effects on the cell viability levels of THP-1, MM6, HL-60, and JurkatE6.1 cells at the range of 1–100 µM concentrations. However, Ctn and Ctn_15-34_ showed similar cytotoxicities on Hela S3 cells. The Ctn_15-34_ sequence could be a better choice for some cancer cells due to its easier production and higher serum stability compared to the full sequence. The most significant point is that the Ctn_1-14_ and Ctn_15-34_ showed lower toxicity in fibroblasts (Falcao et al. 2015).

Cathelicidins are another class of active peptides of snake origin. A K16-substituted 30 amino acid-long peptide KFFRKLKKSVKKRAKKFFKKPRVIGVSIPF derived from *Bungarus fasciatus* was found to have anticancer effects against several cancer cells. The lowest effects of the peptide were on HepG2, MCF-7, and A375 cells with IC_50_ values of 35.7, 50.8, and 70.3 µM, respectively. Its IC_50_ values in H460, PC-3, Lewis, and B16F10 cells were 16.5, 12.5, 10.5, and 7.3 µM, respectively. The anticancer efficacy was linked with membrane channel formation and genomic DNA binding (Tian et al. 2013).

Due to the high cost of snake venom, obtaining lower molecular weight peptides of venom origin by using bacterial expression can be a more suitable option. Their high cost-effectiveness compared to proteins can provide them with advantages. Combining low molecular weight peptides with anticancer potential and those possessing cell-penetrating ability can enhance therapeutic outcomes. Lower molecular weight peptides derived from snake venom represent a class of molecules that ease the discovery of small molecular conventional drugs. However, the above-discussed data reveal only micromolar effective cytotoxic doses of the peptides of venom origin against various cancer cells. Therefore, the above-mentioned systemic approaches are needed to achieve significant results with these lower molecular peptides as well.

## Fractionation and purification

The complexity of snake venoms, arising from the synergistic interplay of dozens of enzymatic and non-enzymatic proteins, necessitates a robust fractionation strategy to deconvolute individual bioactive components. Despite the rich arsenal, the translation of venom toxins to the clinic is hampered by interrelated barriers: most toxins require micromolar concentrations for efficacy (1), venom compositions vary among species and seasons (2), and systemic toxicity limits dosing. Therefore, fractionation and purification of toxins are needed to overcome the above barriers. In this section, we discuss features relevant to fractionation and purification that can contribute to enhancing the anticancer potential of snake venom polypeptides.

Effective separation and purification of venom components are essential for identifying bioactive compounds. The complexity of snake venom composition complicates the study of the mechanism of action of the components as biologically active substances. Therefore, the separation of the compound(s) of interest is an unavoidable part of the study of snake venoms. The main technique used so far for this purpose is chromatography: ion-exchange (IEC), size-exclusion (SEC), affinity (AC), and reverse-phase high-performance liquid chromatography (RP-HPLC) (Sahyoun et al. 2022). Hydrophilic interaction liquid chromatography (HILIC) – a novel type of chromatography, has started to be utilized in venomics in recent years (Falla et al. 2025). Gel electrophoresis is also a widely used method for the separation of proteins according to their pI values in snake venoms. OFFGEL electrophoresis, a new approach in electrophoresis, is a very suitable method to collect the fractions in a buffer solution that overcomes solubility and recovery issues (Abd El-Aziz et al. 2020). For the first steps, gel filtration and ion exchange chromatography methods are used, depending on the aim. Further purification can be carried out by using HPLC for individual peptides. Mass-spectral analyses or Edman degradation are used for structure establishment (El Hakim et al. 2011, Ye et al. 2013). Since all these approaches have pros and cons (Table 2), one or some of them can be used in a certain combination regarding the research objective. Ultrafiltration is one of the efficient methods for the fractionation of biomolecules. The application of this method can make a promising contribution to the fractionation of snake venoms.

Snake venom and its distinct substances have been established to show anticancer efficacies in ng/mL (Kerkkamp et al. 2018) or µg/mL (Izidoro et al. 2014) concentrations in various cancer cells. Various ratios of anticancer property-revealing molecules in various snakes can explain the main reason for these high differences. These discrepancies can be linked to varying ratios of toxin molecules across different snake species. For example, the transcriptomic analyses of *Micrurus mipartitus* showed the dominance of 3FTx, CTL, metalloproteinases, and KSPIs over lipases, VEGF, phospholipases, and other minor families (Saldarriaga-Córdoba et al. 2024). Therefore, fractionation and purification are essential to identify more and less toxic molecules as major and minor classes of substances. These approaches are fundamental to advancements in this field. Since similar responses in biological systems may not be expected due to variations in the venom compositions depending on seasons and geographical locations, fractionation can at least be effectively used to define more cytotoxic fractions that could be utilized to isolate and purify more potential polypeptides. Several works dealt with the comparison of yield- and activity-related features of distinct molecules. (Vivas-Ruiz et al. 2023) reported the expression levels of four snake toxins. Pictolysin-III and LAAO were similar; pictobin was at lower concentrations, whereas phospholipase A2 was in higher amounts compared to them (Vivas-Ruiz et al. 2023).

(Sadat et al. 2023) fractionated the venom of the *Naja naja oxiana* cobra by using size-exclusion chromatography. Two fractions holding polypeptides with MW < 11 kDa, shown by gel electrophoresis, showed several-fold higher efficacies in HT-29 cells over HEK-293 human embryonic kidney cells. The partial sequence with a 7 kDa MW was found to belong to neurotoxin II. The EC_50_ values of the polypeptide were 0.7 and 8 µg in HT-29 and HEK-293 cells, respectively (Sadat et al. 2023). (Latinović et al. 2017) isolated a disintegrin having an MW < 15 kDa by gel filtration and RP-HPLC from the *Vipera ammodytes ammodytes* venom. A 0.5 µg of polypeptide caused significant reductions in MDA-MB-231 cell viability levels (Latinović et al. 2017).

A critical review of fractionation and purification methods reveals the absence of a technique applicable to all venom toxins. The physicochemical properties of the target molecule might include its MW, isoelectric points, amphiphilicity/hydrophobicity/hydrophilicity, and post-translational modifications. These features should dictate the purification workflow. For instance, SEC is a gentle approach and preserves native conformations of polypeptides (Zhai et al. 2025), making it suitable for enzymes, whose enzymatic activity is essential for their anticancer function. However, it offers limited resolution for similarly sized proteins, which may prevent the fractionation of those falling within a close MW range (Gritti 2023). Conversely, RP-HPLC provides high resolution and is indispensable for separating hydrophobic peptides and isoforms (Aguilar and Hearn 1996); its reliance on organic solvents and low pH can cause the irreversible denaturation of enzymes (Büyükköroğlu et al. 2018), which prevents its utilization for them. These two points, including resolution and preserving bioactivity, are a recurring challenge in venom fractionation. Therefore, multi-dimensional strategies need to be developed to provide a well-designed resolution by preserving Advanced chromatographic and electrophoretic techniques are critical for the isolation of potent anticancer toxins. Effective fractionation and purification techniques are essential for further development in this discipline. Utilizing the advances of chromatography and electrophoresis can lead to the identification of the most effective anticancer means in the venom, especially among the minor components.

## Delivery systems

The successful translation of snake venom-derived polypeptides into clinically viable anticancer agents depends not only on their intrinsic potency and selectivity but also on their ability to reach target tissues in sufficient amounts by avoiding systemic toxicity. As discussed in the preceding section, purification can enable obtaining highly active toxins. However, even the most potent venom-derived peptides and proteins, like any other polypeptide drug, face various barriers, including proteolytic degradation, immunogenicity, and poor tumor penetration that limit their therapeutic utility (Asrorov et al. 2020, Asrorov et al. 2022). Several advanced delivery systems have emerged to enhance the therapeutic efficacy of venom-derived anticancer agents. In this section, we objectively evaluate delivery platforms investigated for venom toxins, which include NPs, cell-penetrating peptides, liposomes, and hydrogels, with particular attention to their mechanisms of action, preclinical efficacy, and limitations.

Nanotechnology and targeted delivery systems can improve the therapeutic index of the venom-derived peptides. The delivery of biologically active molecules to targeted tissues using various systems is one of the most effective approaches to enhance their drug potential by reducing passive transportation (Kalambhe et al. 2026). So far, several studies have focused on enhancing the efficacy of these snake venom components through various delivery systems. In one simple approach, NKCT1, a 6.7 kDa MW polypeptide isolated from *Naja kaouthia*, was reported to be an effective prodrug when tagged to gold NPs. The tagged peptide inhibited MCF-7 breast cancer cells via cyclin-dependent cell cycle arrest. A 3.9 µg/mL concentration of peptide significantly inhibited the viability level of MCF-7 cells but not MDA-MB-231 cells. The results were attributed to the expression of estrogen receptors (Bhowmik and Gomes 2017). These and other results explain the significance of delivery systems. In this section, we discuss the enhanced efficacies of active venom polypeptides that result from their delivery.

### Silica NPs

Among the diverse array of delivery platforms, nanoparticle-based systems, including polymeric nanoparticles, solid lipid nanoparticles, and inorganic nanocarriers, have garnered considerable attention for their ability to improve the pharmacokinetic profile and tumor targeting (Yao et al. 2020). Silica NPs are very efficient drug-delivering molecules that have been established to be useful in combating cancer (Yousefiasl et al. 2025). (Badr et al. 2013) loaded the *Walterinnesia aegyptia* venom into silica nanoparticles, doubling its efficacy. The venom IC_50_ values in both PCa and PC3 prostate cancer cells were 5 µg/mL, and by loading into silica NPs, these levels were halved in both cases. Besides, significant reductions were observed in the cell proliferation levels of PCa, PC3, and LNCaP cells after loading into NPs (Badr et al. 2013). In another interesting work, the snake venom of *Walterinnesia aegyptia* was loaded into silica NPs to enhance its efficacy. Both the venom and the loaded venom resulted in day-dependent changes in tumor volume. For 38 days of treatment, loading venom in silica NPs inhibited the multiple myeloma tumor volume threefold more effectively compared to free venom. Besides, significant reductions were observed in cell proliferation and migration levels. Moreover, mitochondrial membrane potential was halved by loading, and caspase-3, –8, and –9 levels more than doubled. Significant enhancements in ROS and hydroperoxide levels were observed upon loading. The authors concluded that the efficacy of the approach led to apoptosis (Al-Sadoon et al. 2013). *Walterinnesia aegyptia* venom loaded in silica NPs, resulting in enhanced anticancer efficacy in RPMI 8226 and U266 multiple myeloma cells, the cell viability levels of which were reduced significantly. The approximate 25 µM IC_50_ of the venom was reduced by 10 µM by encapsulating it into NPs. Molecular analysis proved the efficacy of loading polypeptides in silica NPs (Badr et al. 2012).

### Cell-penetrating functions

Cell-penetrating peptides facilitate the intracellular delivery of drugs and, therefore, are effectively utilized in anticancer strategies (Asrorov et al. 2023). Some polypeptides isolated from snake venom were found to possess a cell-penetrating function. Crotamine is one of those peptides, the cell-penetrating efficacy of which was determined. It is a 42-amino-acid-long molecule with a YKQCHKKGGHCFPKEKICLPPSSDFGKMDCRWRWKCCKKGSG sequence derived from a rattlesnake, which was found to have three cystine bonds that provide stability to the molecule (Tansi et al. 2019). By the amino acid sequence, crotamine shows a high ratio of lysine (K) that could be classified as a lysine-rich CPP (Asrorov et al. 2023). The peptide isolated from *Crotalus durissus terrificus* was reported to bind to DNA or RNA and function as a delivery system. Linking crotamine with PEGylated gold NPs enhanced the internalization of the gold NPs (Hayashi et al. 2020). These results can support the utilization of this peptide for the development of new recombinant polypeptides. On the other hand, nine lysine and two arginine residues cause the formation of a high polarity in the molecule. Besides, the peptide was reported to have cell-penetrating ability (Rádis-Baptista and Kerkis 2011). Its 1 µg/mL concentration almost halved the viability of B16F10 cells; a 5 µg/mL concentration reduced that level by 75%. Its impact on Mia-PaCa-2 cells was not as effective as on B16F10 cells. Similar concentrations reduced the cell viability level by ~30 and 50%, respectively (Pereira et al. 2011). The locations of various amino acids on the surface of venom toxins were reviewed as the main factors defining their toxic level. The higher ratio of amino acids with a more hydrophobic nature or lipophilicity can be one of the major factors determining the penetration efficacy and, thus, cytotoxicity (Dubovskii et al. 2014). Therefore, including cell-penetrating peptides while constructing recombinant peptides or proteins can be a crucial point in enhancing their cytotoxic effects (Asrorov et al. 2023). Incorporating these peptides into therapeutic designs enhances drug uptake and efficacy.

### Liposomes

**Fig. 10:**
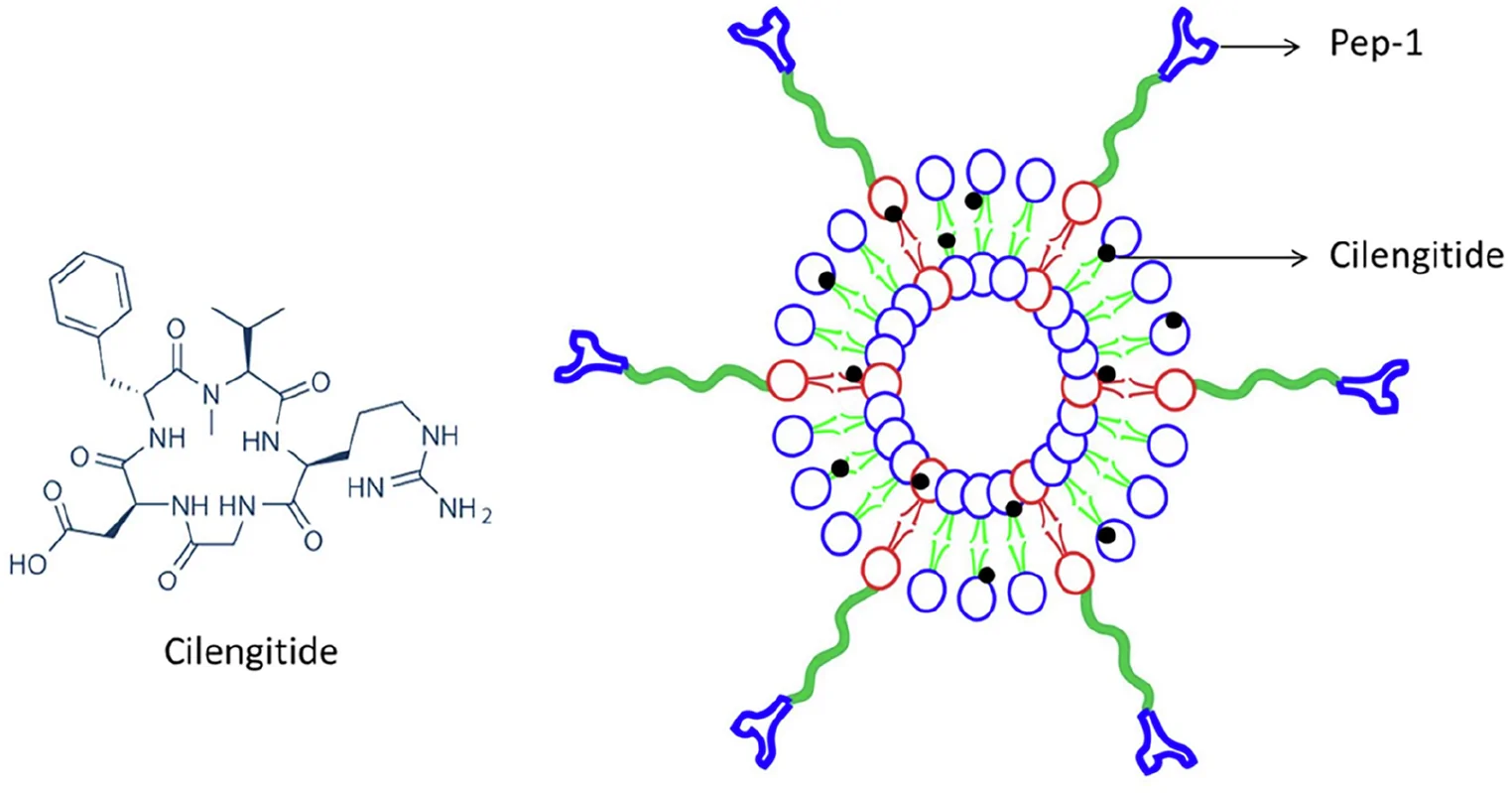
Loading cilengitide in liposomes, the surface of which was modified with Pep-1 peptide. The figure has been reused from (Jiao et al. 2017) with the permission of Elsevier

Liposomal encapsulation represents one of the most practically validated nanocarrier strategies that reduce systemic toxicity (Maroof et al. 2026), while enhancing passive tumor accumulation (Kalra et al. 2024). Liposomes are one of the most practical drug delivery systems that improve pharmacokinetics and targeting. Findings show the efficacy of liposomes that can be controlled effectively to target tumors (Tu et al. 2020, Asrorov 2022). In one work, loading cilengitide in liposomes did not lower the viability levels of U87 cells. However, the surface modification of liposomes with Pep-1 peptide resulted in a significant improvement in the drug efficacy (Fig. 10). Drug loading in surface-modified liposomes reduced the drug IC_50_ value from 6.85 to 2.38 µg/mL. Compared to the effects of the free drug, loading and targeting it using liposomes halved the tumor volume in mice bearing U87 cells (Jiao et al. 2017).

(Kim et al. 2016) co-loaded salmosin (pSal), a snake venom-derived disintegrin, and pDNAs of the IL-12 (pIL12) in PEG-modified cationic liposomes (pSal+pIL12/PEG-lipoplex). The developed system inhibited tumor growth by a third volume in mice bearing SK-OV-3 cells. The DOX combination with pSal +pIL12/PEG-lipoplex resulted in a synergistic effect and inhibited tumor growth several-fold more effectively. The formation of an immunolipolex holding anti-EGF receptor (pSal+pIL12 immunolipoplex) was found to be a more optimal option against the SK-OV-3 tumor. It inhibited tumor growth more than threefold compared to the control. The combination of pIL12+pSal immunolipoplex with DOX completely inhibited tumor growth for 22 days of treatment (Kim et al. 2016). These results enable us to suggest that various combinations with practically available anticancer drugs can cause synergistic effects. Biomimetic delivery systems composed of blood cell membranes have been established as more effective delivery means compared to liposomes. Despite the complexities of preparation and obtaining them, they have advantages such as avoiding immune attacks (Asrorov et al. 2021). In another work, a peptide termed CDX with a sequence FKESWREARGTRIERG derived from candoxin of snake venom origin (Nirthanan et al. 2002) was incorporated into red blood cell NPs (RBCNPs) to deliver DOX to tumor cells (RBCNPs/DOX). Targeting RBCNPs/DOX NPs with CDX enhanced (CDX-RBCNPs/DOX) in vivo activity of the developed NPs, CDX-RBCNPs/DOX extended the total and 50% animal survival rates of glioma-bearing mice by three and four days, respectively (Chai et al. 2017). Liposomal formulations improve the pharmacokinetics and targeting of snake venom; however, establishing drug loading capacity and efficiency might be complex.

### Hydrogels

Hydrogels offer highly biocompatible three-dimensional systems that efficiently encapsulate both small and macromolecular therapeutics, providing them with sustained release and controlled release at the tumor site (Segneanu et al. 2025). They are characterized by their oral, subcutaneous, and intramuscular administration (Mehta et al. 2023). Hydrogels, providing sustained release and local delivery, can be an effective therapeutic to prevent metastasis when used for local administration (Li et al. 2023). Loading snake venoms in hydrogels made of synthetic polymers has been proven an efficient approach, leading to a prolongation effect of at least (Hamzaoui and Laraba-Djebari 2021). In this aspect, PLGA is a reliable option that could be combined with other biopolymers or synthetic polymers and is biocompatible (Santos-Silva et al. 2022). The electrostatic interactions between the carboxylic group of PLGA and the amino group of loaded polypeptides provide extra stability to the formed systems (Huang et al. 2022). (Zhang et al. 2012) loaded the cobra toxin in PLGA microspheres and in a mixture with ricin co-loaded in PLGA-PEG-PLGA hydrogels. The release of venom toxin was smaller than that of ricin due to its loading in PLGA. The prolongation effect was observed in the toxin efficacy (Zhang et al. 2012). These results explain the significance of loading in polymers to enhance their efficacy.

Incorporating venom-derived proteins and peptides with innovative delivery platforms such as nanoparticles, liposomes, or hydrogels is key to translating venom peptides into clinical use. Delivery systems can enhance their efficacy, which can be attributed to their higher stability, prolongation effect, targeting ability, etc. In vivo experiments can prove their further effectiveness. However, loading higher MW compounds in various delivery systems requires optimization of various factors.

In summary, delivery systems play the role of a bridge between efficient in vitro cytotoxicity and meaningful in vivo antitumor activity. The evidence reviewed in this section shows that encapsulation in nanoparticles, liposomes, or hydrogels, as well as conjugation with cell-penetrating peptides, can significantly enhance the stability, tumor targeting, and intracellular uptake of venom toxins, often reducing effective doses by several-fold and improving survival outcomes in animal models. However, the above-discussed data is very is not sufficient in terms of claiming the real effectiveness of the developed systems with those used toxins/venoms. Moreover, the complexity of formulations, batch-to-batch reproducibility, and the potential for altered bioactivity upon conjugation remain main hurdles due to limited amounts of toxins and issues in their purification.

## Cilengitide

Several polypeptides derived from snake venom have reached clinical studies or clinical use. (Waheed et al. 2017) reviewed those therapeutics that originated from snake venom (Waheed et al. 2017). Among them, echistatin, belonging to disintegrins (Tome et al. 2016), and moojenin, belonging to metalloproteinases (de Morais et al. 2012), were reported among those that reached clinical use as antithrombotic means. Some other snake venom-derived therapeutics were also reported as thrombolytic or anticoagulant means (Waheed et al. 2017). In another recent review, Almeida et al. reviewed peptides as anticancer candidates (Almeida et al. 2025). They report 22 FDA-approved peptide therapeutics, published from 1985 to 2024, used mainly for hormone-related cancers. Recent advances exemplify cilengitide, a synthetic peptide originating from the disintegrin motif, as the molecule that reached clinical studies.

Among all snake venom-inspired anticancer agents, cilengitide is one of the few molecules to have advanced into extensive clinical evaluation. This synthetic cyclic pentapeptide [-Arg-Gly-Asp-N_met_Val-Phe-], having an RGD sequence, derived from disintegrins, was rationally designed to antagonize integrins αvβ3 and αvβ5 receptors that play significant roles in angiogenesis, tumor cell adhesion, and metastasis. In this section, we discuss the preclinical and clinical outcomes of research around cilengitide.

### in vitro and in vivo efficacies of cilengitide

Cilengitide, a synthetic RGD peptide inspired by snake venom disintegrins, has progressed to clinical studies as an integrin antagonist. The development of peptide-based cytotoxic compounds led to the synthesis of some synthetic peptides holding an RGD sequence. Among these synthetic peptides, cilengitide deserved greater attention and reached clinical stages. This cyclic pentapeptide was found to bind αvβ3 and αvβ5-ligand integrins (Hegemann et al. 2014). Its 20 µM concentration reduced the cell adhesion level of MDA-MB-231 cells by ~70%. Its effects on cell migration levels were clearer; 20 nM of the peptide halved the cell migration of MDA-MB-231 cells. Besides, cilengitide reduced pre-osteoclast adhesion to the matrix holding RGD. The authors concluded that it interferes with αV integrins (Chillà et al. 2015). (Zhang et al. 2019) report that cilengitide could significantly reduce the viability levels of SCC4, SCC15, and SCC25 cells at only 100 µM concentrations. Similar effects were observed in Tu57 and Tu63 primary culture cells at 12.5 µM. These results explain the very low effects of cilengitide on the above HNSCC cells (Zhang et al. 2019).

Cilengitide has been used in combination with therapeutics or ionizing radiation. Past and ongoing studies imply its utilization for cerebral tumors due to its high efficacy in targeting αvβ3 and αvβ5 (Leblond et al. 2011). Cilengitide was also determined to suppress malignant pleural mesothelioma (MPM) since it expresses integrins αvβ3 and αvβ5 that are effectively targeted by it. The authors suggested the contribution of cilengitide to the invasiveness of MPM cells, which aligned with cell growth suppression (Cheng et al. 2014).

A comparative study shows a significantly higher efficacy of cilengitide over 5-FU and GRGDSp peptide. The authors concluded that cilengitide has a higher efficacy than other RGD peptides due to its affinity to integrins. Identifying the quantity of cilengitide-binding integrins could support its predictive effects on various cancer cells. In Hep-2 laryngeal cancer cells, it induced caspase-3, leading to apoptosis (Wang et al. 2014). (Martin et al. 2014) report that the combined effects of IL-12 and cilengitide extended the animal survival rate compared to those treated with IL-12 alone without cilengitide. The combination showed an additive effect on murine hematogenous brain metastases (Martin et al. 2014). In another study, PET was concluded to be useful for assessing the effects of cilengitide in rats bearing breast carcinoma metastases (Cheng et al. 2011).

Targeting integrins by cilengitide and EGFR by cetuximab did not result in a synergistic effect on head and neck squamous cell carcinoma (HNSCC) cells ex vivo (Wichmann et al. 2017). However, the combination of cilengitide and cisplatin resulted in a synergistic effect on three HNSCC cells – SCC25, CAL27, and FaDu. Despite the low efficacy of cilengitide on cell survival rates, its combined effect at 10 µM with 1–10 µM cisplatin showed similar linear changes in the survival rates. Synergistic effects of cilengitide were also observed in combination with radiotherapy. These results imply studying the combined effects with other anticancer agents that could enhance its therapeutic efficacy (Heiduschka et al. 2014). Due to its nanomolar efficacy against cancer cells, cilengitide reached clinical trials (Table 3) (Weller et al. 2016). Cilengitide was found to synergistically enhance the anticancer effects of vasculostatin-expressing oncolytic virus against glioma cells (Fujii et al. 2013).

### Clinical studies of cilengitide

Clinical trials of cilengitide have evaluated its safety and efficacy across various cancer types. We discussed in other sections that snake venoms and distinct components have µg/mL or micromolar IC_50_ values in various cancer cells, respectively. This is probably one of the main reasons why protein and polypeptides of snake venom origin have not reached medical practice as pure compounds. In our previous review papers, we discussed protein drugs or prodrugs that reached clinical studies (Asrorov et al. 2022). They revealed positive outcomes at nanomolar concentrations (Asrorov et al. 2020, Asrorov et al. 2022). On the other hand, natural anticancer compounds that have nanomolar effectiveness in cancer cells have yet to reach clinical studies due to their side effects (Asrorov et al. 2023). The progression of cilengitide to phase II and II clinical trials represents a landmark achievement for the field, demonstrating that venom-derived structural motifs can be successfully translated into drug candidates. However, the clinical trajectory of cilengitide has been characterized by mixed outcomes. Our search for ‘clinical studies of snake venom and the components’ in NCBI resulted in fewer than fifteen papers, many of which were devoted to cilengitide, with an RGD sequence targeting integrins. Even if cilengitide is a cyclic synthetic peptide having [-Arg-Gly-Asp-N_met_Val-Phe-] sequence, due to its common integrin-targeting property as snake venom disintegrins and derived from the RGD sequence of disintegrins, we reviewed the clinical outcomes of this peptide. Cilengitide passed various stages of clinical studies against various cancers, including solid tumors. Its antiangiogenic activity is considered to be the main contributing factor to the results of clinical studies. In most clinical studies, 500–2000 mg dose ranges were utilized. Some cases resulted in linear pharmacokinetic parameters in patients (Reardon et al. 2011). This dose was well tolerated in patients with prostate cancer, which supports the integrin inhibition (Bradley et al. 2011).

Herein, we provided the outcomes of those thirteen clinical trials in a table format (Table 3). In general, the addition of cilengitide did not remarkably improve the therapeutic effects of chemotherapeutic agents. The authors concluded that further studies are required. In other cases, the studies were discontinued due to the low efficacy of cilengitide with other drugs (Table 3).

Cilengitide demonstrates the translational potential of venom-inspired integrin-targeting peptides. Despite various outcomes in clinical studies, the results of combined drug administrations can serve to identify further structure-guided design in cancer therapy.

In conclusion, cilengitide shows the outcomes of venom research that reached clinical studies. Its progression into clinical trials validated the fundamental premise that snake venom disintegrins and the RGD motif in particular can serve as a scaffold for drug development. However, the obtained results imply that improvements are a prerequisite. The structural insights gained from cilengitide, particularly its cyclic conformation, which enhances stability and specificity, continue to guide the rational design for novel disintegrin-inspired peptides. As we move to the next section, we will examine how secondary and tertiary structures and design principles, drawing on lessons from cilengitide and other venom peptides, can be leveraged to optimize the selectivity, stability, and potency of future generations of venom-inspired therapeutics.

## Secondary and tertiary structures of venom polypeptides

The structural diversity of snake venom polypeptides fundamentally determines their mechanism of action, target selectivity, and therapeutic potential. Unlike small-molecule drugs that rely on simple binding interactions (Tabana et al. 2023), venom toxins exploit complex three-dimensional architectures arising from their affinity for key biomolecules of the host organism (Walkinshaw et al. 1980; Ullah and Masood, 2020). While the preceding sections focused on functional, pharmacological, and clinical outcomes, this section provides a comparative structural analysis of representative venom toxin families – disintegrins, PLA2, LAAO, and 3FTxs, using the digital data provided in the open-access Protein Data Bank. Understanding these structural features is essential not only for expanding observed biological activities but also for guiding the rational design of improved therapeutic agents. We discuss some features of the secondary and tertiary structures of the selected polypeptides in terms of their stability, amphiphilicity, charge-holding properties, and related points.

**Fig. 11:**
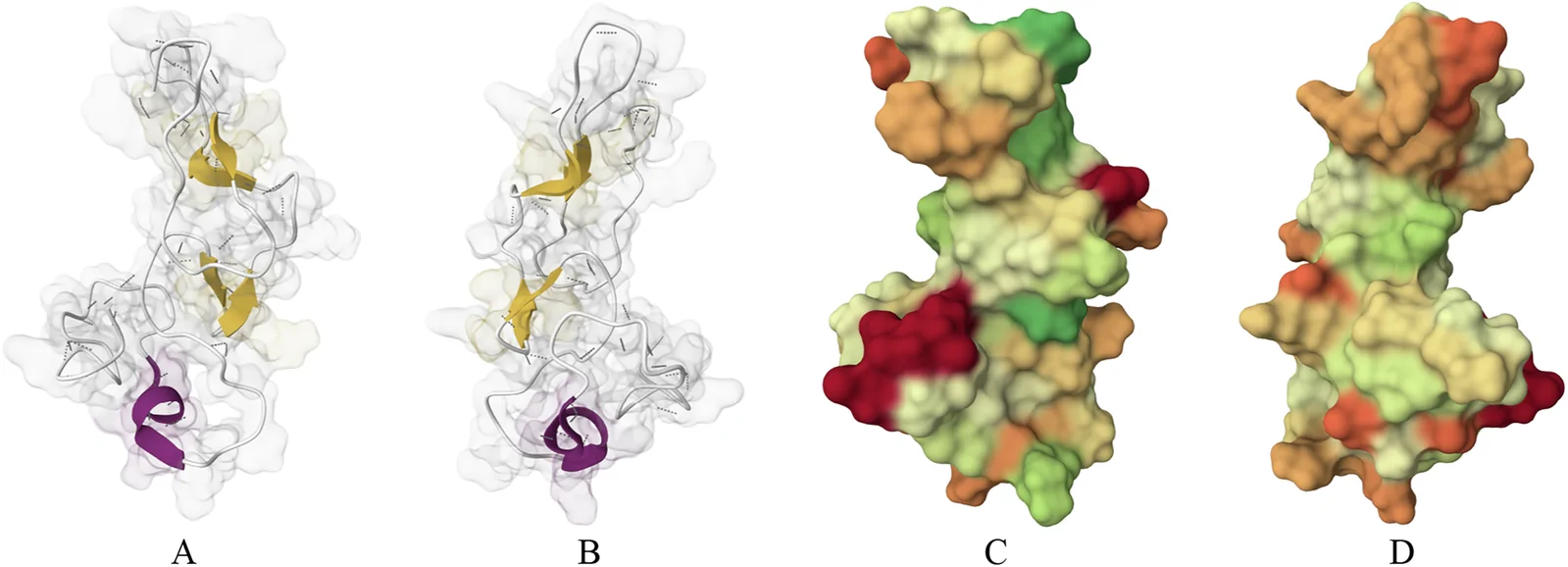
The secondary and tertiary structures of jarastatin disintegrin. The structures were developed online in the Protein Data Bank https://www.rcsb.org/3d-view/8S9E. Figures **A** and **B** show α-helices and β-sheets in different colors. The dotted lines show non-covalent interactions in the molecule. Figures **C** and **D** show the distribution of hydrophobicity and hydrophilicity of the surface of the protein, as well as charged amino acid residues. We used *Cartoon*, *Gaussian volume*, *Non-covalent interactions*, and *Secondary structure* options to generate the secondary structure of the polypeptide. The *Molecular surface* and *Hydrophobicity* options were used to develop a three-dimensional structure of the polypeptide. Dark colors (maroon, brown, rust, goldenrod) show highly hydrophilic amino acid residues, including Glu, Asp, Lys, Arg, Gln, and Asn. Green colors of various intensities show the locations of Phe and Tyr (dark green), Leu (light green), and Ile (pale green). Other colors around pale yellow and banana show the remaining amino acids

Disintegrins, containing RGD or other motifs, are the first example to discuss in this section. Earlier, we mentioned the diversity of disintegrins, differing from each other by the MW and the number of disulfide bonds rigidifying motifs. In Fig. 11, we demonstrated the secondary and tertiary structures of jarasattin as an example of disintegrins. We observe only one small α-helix and four β-sheets together with a small number of non-covalent interactions, which might explain the flexibility of the molecule consisting of 73 amino acid residues.

The predominance of β-sheets over α-helices, combined with a smaller number of non-covalent interactions, might explain the conformational plasticity of jarastatin. The flexibility is critical for its ability to adapt to the dynamic binding pockets of the integrin subtypes (αvβ3, αIIβ3), enabling the RGD motif to be optimally oriented for high-affinity recognition. However, the structural flexibility may also contribute to off-target binding, which might increase its interactions with various isoforms.

**Fig. 12:**
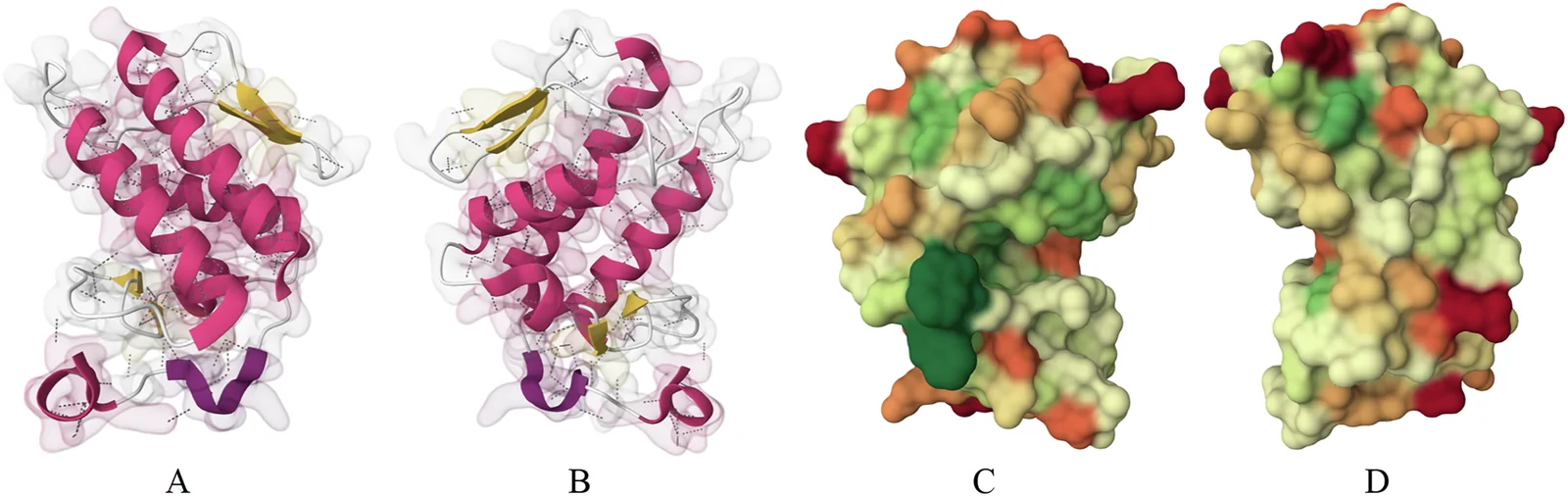
Secondary and tertiary structures of PLA2. The structures were developed online in the Protein Data Bank https://www.rcsb.org/3d-view/1M8T. Figures **A** and **B** show α-helices and β-sheets in different colors. The dotted lines show non-covalent interactions in the molecule. Figures **C** and **D** in the second horizontal line show the distribution of hydrophobicity and hydrophilicity of the surface of the protein, as well as charged amino acid residues. Colors stand for similar amino acids as described in Fig. 11

In Fig. 12, the secondary and tertiary structures of PLA2, consisting of 119 amino acid residues, are shown. Four α-helices and a high number of non-covalent interactions imply high stability of the molecule. The three-dimensional structure shows the hydrophobic site around the pocket. Most probably, the hydrophobic pocket is responsible for its interaction with membrane lipids (Li et al. 2026).

The abundance of α-helices and extensive non-covalent interactions confer remarkable structural stability to PLA2, which is essential for maintaining its catalytic function in the extracellular environment. The prominent hydrophobic pocket can be responsible for its membrane-disrupting activity, enabling the enzyme to insert into the phospholipid bilayer and hydrolyze fatty acids. The high density of positively charged residues (Lys and Arg) on the surface surrounding the hydrophobic pocket creates an amphiphilic character that might enhance the selectivity of PLA2 isoforms for cancer cell membranes over normal cells due to their higher densities of charges on their surface (Chen et al. 2016).

**Fig. 13:**
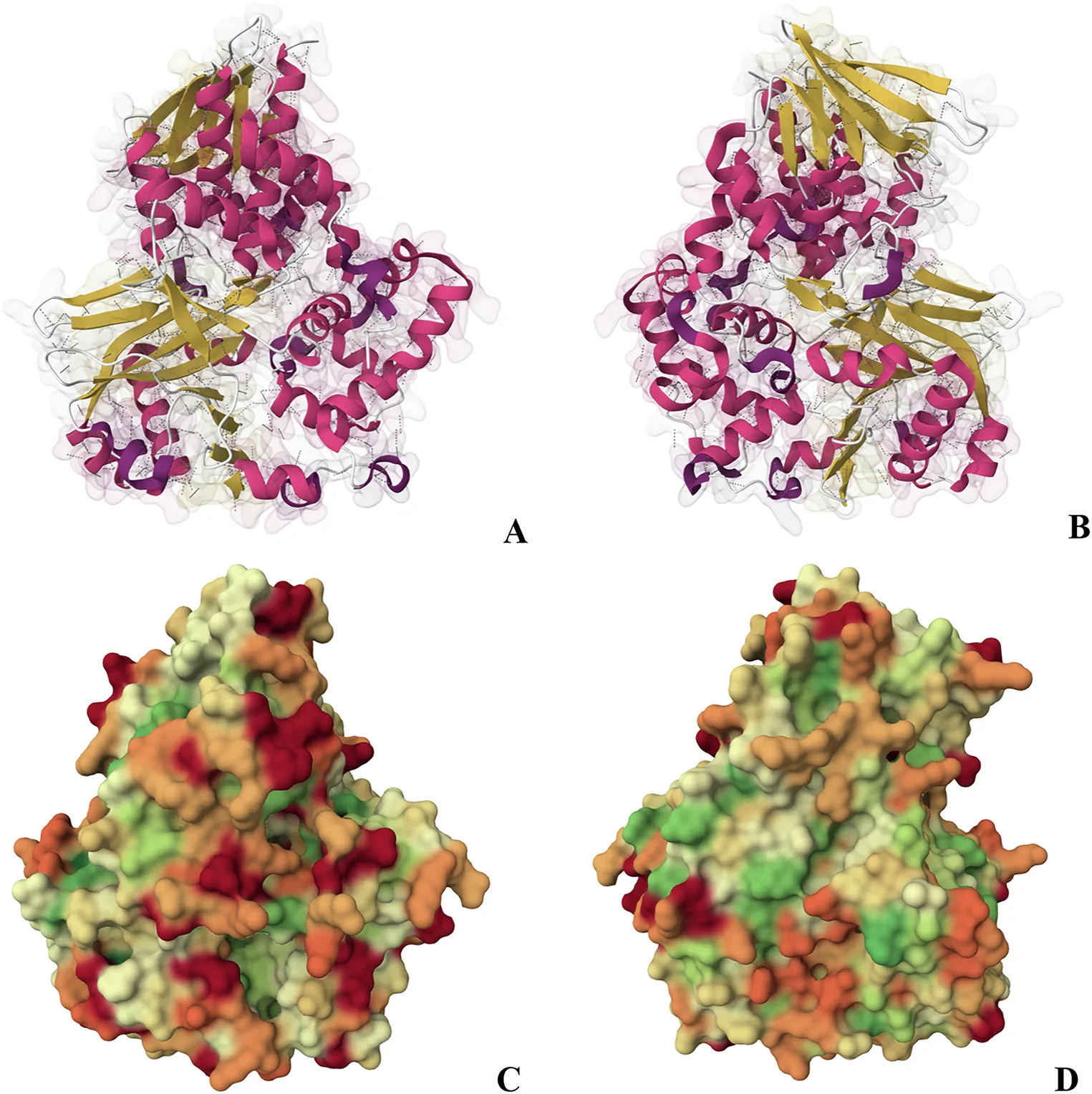
Secondary and tertiary structures of LAAO. The structures were developed online in the Protein Data Bank https://www.rcsb.org/3d-view/1TDN. Figures **A** and **B** show α-helices and β-sheets in different colors. The dotted lines show non-covalent interactions in the molecule. Figures **C** and **D** in the second horizontal line show the distribution of hydrophobicity and hydrophilicity of the surface of the protein, as well as charged amino acid residues. Colors stand for similar amino acids as described in Fig. 11

Figure 13 shows the secondary and tertiary structures of LAAO, consisting of 486 amino acid residues. Figure 13A and B imply high stability of the protein, provided by a high number of α-helices, β-sheets, and non-covalent interactions. Based on the high ratio of charged amino acid residues, such as glutamic acid, aspartic acid, arginine, and lysine on the surface of LAAO, we can suggest its high water solubility (Trevino et al. 2007). A high number of hydrophobic cores on its surface, as described in Fig. 13D, might indicate its involvement in many interactions with amphiphilic molecules (Lillo et al. 2018, Kar 2025). Highly conserved negatively charged sites on the surface, described in Fig. 13C, imply their probable interaction with positive ion charges (Ullah 2020).

The high proportion of charged residues (Glu, Asp, Lys, and Arg) on the surface of LAOO explains its high water solubility and suggests that the enzyme functions primarily as a soluble, extracellular generator of H_2_O_2_, rather than as a membrane-interacting protein. The highly conserved negatively charged patches might mediate interactions with divalent cations (Jing et al. 2018) that might modulate LAAO activity and stability. From a translational relevance, the tertiary structure suggests a diffusion-limited feature of LAAO due to high level of charged groups. Therefore, utilization of delivery systems that localize the enzyme to the tumor microenvironment can effectively enhance its anticancer efficacy.

**Fig. 14:**
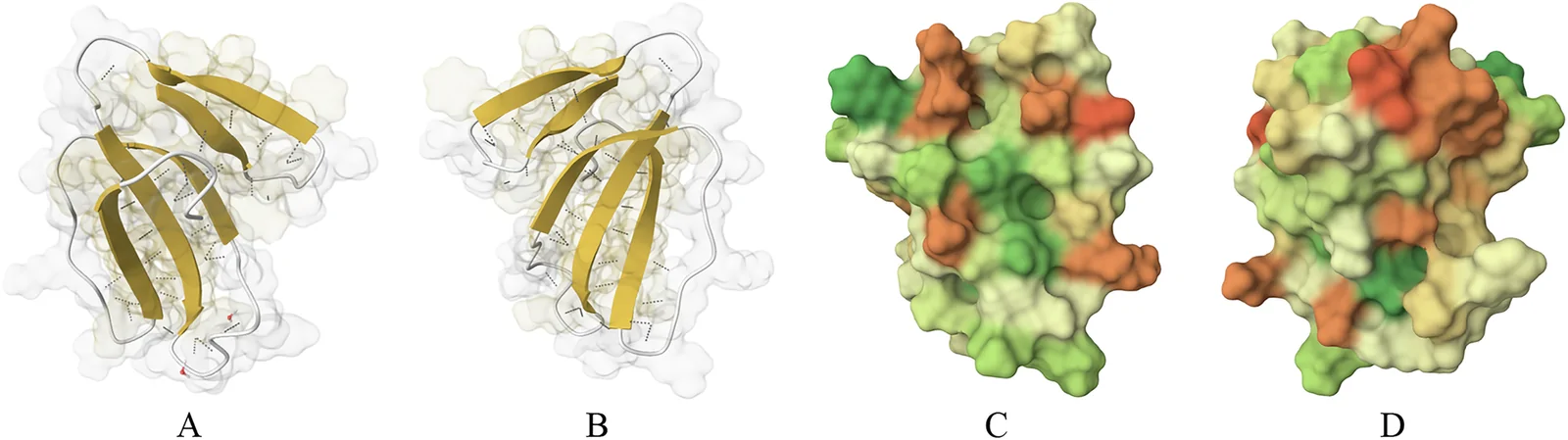
Secondary and tertiary structures of cytotoxin II from *Naja naja Oxiana*. The structures were developed online in the Protein Data Bank https://www.rcsb.org/3d-view/1CB9. Figures **A** and **B** show α-helices and β-sheets in different colors. The dotted lines show non-covalent interactions in the molecule. Figures **C** and **D** in the second horizontal line show the distribution of hydrophobicity and hydrophilicity of the surface of the protein, as well as charged amino acid residues. Colors stand for similar amino acids as described in Fig. 11

The secondary and tertiary structures of cytotoxin II, consisting of 60 amino acids, more hydrophobic nature than the other above-discussed polypeptides. Figure 14A and B show five β-sheets with no α-helix that might imply a high flexibility of the molecule. The surface, described in Fig. 14C and D, shows a small ratio of hydrophilicity of the molecule with several hydrophobic pockets that are crucial for recognition and binding of ligands and substrates (Lai et al. 2024).

The absence of α-helices and the exclusive reliance on β-sheets, stabilized by a high number of non-covalent interactions, can provide the molecule with a moderate rigidity. We can suggest a moderate flexibility of the molecule based on the absence of an α-helix. The hydrophobic pockets observed on the surface can be structural determinants of membrane insertion, a common feature for 3FTxs (Dubovskii et al. 2005). Deeper and systematic analyses of the 3FTxs might reveal more common structure-activity relationships of the members of this class.

In summary, the structural analyses discussing the secondary and tertiary structures of several isoforms of toxins can clarify their putative efficacy that can be useful their flexibility, stability, amphiphilicity and other relevant features based on experimental data provided in digital form. This approach can ease the selection of isoforms for computer simulation or other *in silico* analyses that can be effectively utilized for protein engineering and accelerate the research towards the identification of optimal venom-derived scaffolds and design of next-generation anticancer peptides with improved therapeutic efficacies.

## Conclusive remarks

In summary, snake venom represents a rich and not sufficiently explored source of bioactive polypeptides with considerable antitumor potential. Through diverse mechanisms, including integrin inhibition, induction of apoptosis, oxidative stress, and disruption of cellular membranes, venom-derived toxins exhibit promising cytotoxic and antimetastatic activities across a range of cancer cell lines. However, their translation into clinically viable therapeutics is hindered by challenges such as efficacy at micromolar range, complex venom composition, and delivery limitations.

Advances in fractionation techniques, recombinant peptide production, and innovative delivery systems such as nanoparticles, liposomes, and cell-penetrating peptides offer pathways to enhance the specificity and stability. While clinical outcomes of venom-inspired agents like cilengitide have shown mixed outcomes, the continued integration of venomics, structural biology, and drug delivery innovation remains a promising avenue for the development of next-generation anticancer therapies.

Based on the evidence reviewed in this work, we highlight the main barriers in snake venom translational research and propose the following priorities for future research:

- Snake venoms are highly diverse in their composition and quantities of major and minor components. Multi-omics venomics studies might be needed to identify major and minor components. Consequently, anticancer efficacies of snake venoms can be primarily evaluated across snake populations.
- Integrins are the largest class among snake venom polypeptides. However, the presence of several motifs complicates their selectivity for anticancer research. Therefore, systematic structure-activity relationship studies are needed to identify a minimal integrin-binding motif with maximal selectivity.
- Many of the venom components revealing potential cytotoxicity are the minor components. Using fractionation and purification processes with the intention of structure establishment can be a more useful action than purifying the polypeptides for their cytotoxicity. Further systematic approaches by obtaining various isoforms of recombinant proteins can ease the identification of the most potent sequences for peptides of snake venom origin.
- Delivery systems have been proven to enhance the cytotoxicity of therapeutic means. For venoms, they may not be suitable due to difficulties in establishing their loading capacities and encapsulation efficacies. However, the efficacies for single components can be determined. Comparisons of delivery platforms such as liposomes vs. NPs vs. CPP-conjugates in the same tumor can specify the efficacies of various platforms.
- Cilengitide is one of the few polypeptides of snake venom origin that have reached clinical stages. However, the clinical results were not satisfactory in many cases. Re-evaluation of cilengitide in biomarker-selected patients (those with high αvβ3 expression) can specify its effectiveness. On the other hand, its utilization in combination with other drugs can lead to synergistic effects that might support its clinical effectiveness.

## Data Availability

Not applicable.
